# Analysis of Hepatic Lentiviral Vector Transduction: Implications for Preclinical Studies and Clinical Gene Therapy Protocols

**DOI:** 10.3390/v17020276

**Published:** 2025-02-17

**Authors:** Peirong Hu, Yajing Hao, Wei Tang, Graham H. Diering, Fei Zou, Tal Kafri

**Affiliations:** 1 Gene Therapy Center, University of North Carolina at Chapel Hill, Chapel Hill, NC 27599, USAwei_tang@med.unc.edu (W.T.); 2 Department of Biostatistics, University of North Carolina at Chapel Hill, Chapel Hill, NC 27599, USA; 3 Department of Cell Biology and Physiology and UNC Neuroscience Center, University of North Carolina at Chapel Hill, Chapel Hill, NC 27599, USA; 4 Carolina Institute for Developmental Disabilities, Carrboro, NC 27510, USA; 5 Department of Genetics, University of North Carolina at Chapel Hill, Chapel Hill, NC 27599, USA; 6 Department of Microbiology and Immunology, University of North Carolina at Chapel Hill, Chapel Hill, NC 27599, USA; 7 Lineberger Comprehensive Cancer Center, Chapel Hill, NC 27599, USA

**Keywords:** lentiviral vectors, gene therapy, Collaborative Cross, preclinical studies, hepatic transduction, intrastrain variations, isogenic phenotypic discordance sleep patterns

## Abstract

Lentiviral vector-transduced T cells were approved by the FDA as gene therapy anti-cancer medications. Little is known about the effects of host genetic variation on the safety and efficacy of the lentiviral vector gene delivery system. To narrow this knowledge gap, we characterized hepatic gene delivery by lentiviral vectors across the Collaborative Cross (CC) mouse genetic reference population. For 24 weeks, we periodically measured hepatic luciferase expression from lentiviral vectors in 41 CC mouse strains. Hepatic and splenic vector copy numbers were determined. We report that the CC mouse strains showed highly diverse outcomes following lentiviral gene delivery. For the first time, a moderate correlation between mouse-strain-specific sleeping patterns and transduction efficiency was observed. We associated two quantitative trait loci (QTLs) with intrastrain variations in transduction phenotypes, which mechanistically relates to the phenomenon of metastable epialleles. An additional QTL was associated with the kinetics of hepatic transgene expression. Genes found in the above QTLs are potential targets for personalized gene therapy protocols. Importantly, we identified two mouse strains that open new directions for characterizing continuous viral vector silencing and HIV latency. Our findings suggest that wide-range patient-specific outcomes of viral vector-based gene therapy should be expected. Thus, novel clinical protocols should be considered for non-fatal diseases.

## 1. Introduction

FDA approval of a gene therapy product as a cancer medication [1] has formally opened a new era in medicine. The successful gene therapy clinical trials that led to the aforementioned FDA policy were premised on lentiviral and gammaretroviral vectors [2,3]. More recently, success in employing adeno associated virus (AAV)-based vectors to treat incurable genetic diseases further underscored the therapeutic potential of gene therapy [4,5]. However, due to safety concerns, the spectrum of gene therapy-treated disorders primarily includes fatal or incurable genetic and acquired diseases.

To fully realize the therapeutic potential of gene therapy, it is essential to minimize patient to patient variations in regards to the safety and efficacy of gene therapy protocols. This requirement cannot be met by current preclinical studies, in which the safety and efficacy of therapeutic viral vectors are mostly evaluated in a large cohort of rodents with an identical genetic background, and contingent with availability, in a small number of large animals (e.g., nonhuman primates). Concerns regarding the reliability of the above preclinical research system for predicting the therapeutic outcomes of viral vector-mediated gene delivery to heterogeneous patient populations were raised by a number of murine-based preclinical gene therapy studies demonstrating strain-specific efficacy and safety [6,7,8]. Indeed, several clinical trials employing different viral vectors reported major adverse effects, which could not be predicted by earlier pre-clinical studies [9,10,11,12,13,14,15,16].

Viral vector-mediated transfer of genetic cargos to target tissues and cells is an orderly process made up of multiple sequential and sometimes overlapping steps, whose completion is required for successful gene delivery. The overall efficiency of this complex multifactorial process is largely determined by the attributes of the parental virus from which a particular viral vector was derived and by a balance of interactions between the virion’s proteins and a plethora of host factors. These host proteins and noncoding RNAs are either required for efficient completion of each step in the viral vector transduction process (dependency factors) or serve as restriction factors, which provide intrinsic immunity and are the first line of defense in the host’s antiviral immune system [17,18,19,20,21]. In a healthy human population and in mouse strains without known genetic deficiencies, the expression and function levels of host dependency and restriction factors are expected to be within a relatively narrow normal range. Thus, mild non-pathologic alterations in the function/level of the abovementioned host factors are expected to significantly affect the outcomes of viral vector-based gene delivery, with minimal adverse effects.

To better understand the effects of host genetic variations on the outcome of lentiviral vector-based gene therapy protocols, we sought to screen for natural allelic combinations that affect the efficacy of hepatic gene delivery by lentiviral vectors in healthy mouse strains. This approach is premised on the ability to associate phenotypic data generated in vivo with specific allelic combinations unique to genetically characterized mouse strains. To this end, we characterized hepatic gene delivery by lentiviral vectors in the Collaborative Cross (CC) mouse strains panel. The CC mouse strains collection is a large panel of recombinant inbred strains, which were derived from a pool of eight genetically diverse inbred mouse strains, including A/J, C57BL/6J (named below as C57B6), 129S1/SvImJ, NOD/ShiLtJ, NZO/HILtJ, WSB/EiJ, PWK/PhJ, and CAST/EiJ. Altogether, the CC mouse strains panel comprises more than 36 million single nucleotide polymorphism (SNP) sites [22]. The highly genetically diverse CC mouse strains have been successfully used for genetic mapping [23,24] and the development of new disease models [25,26,27]. Importantly, in a CC mouse-based pilot study, Suwanmanee et al. [8]. demonstrated major strain-specific differences in hepatic lentiviral vector transduction characteristics, including the level of hepatic transgene expression and the pattern of vector biodistribution.

Here, we report on a large-scale genetic study, in which 254 female mice from the commonly used C57B6 mouse strain and 41 CC strains were intraperitoneally (IP) administered with lentiviral vectors expressing the firefly luciferase cDNA under the control of a liver-specific promoter (hAAT). The efficacy of gene delivery was evaluated by the periodic analysis of hepatic luciferase expression. The vector copy number (VCN) in the liver and spleen tissues served as a surrogate marker of vector biodistribution. A total of 23 traits of hepatic gene delivery by lentiviral vectors were phenotypically and genetically analyzed. As previously described [8], we observed significant heritability across these measures. We located a QTL in chromosome 1, which was associated with changes in hepatic transgene expression between week 1 and week 3 post vector administration (PVA). Four genes in this QTL, *SERTAD4*, *IRF6*, *TRAF3IP3*, and *UTP25*, are involved in epigenetic regulation, development, and the innate immune response and thus can be considered as candidate genes contributing to the above phenomenon. In addition, we identified two QTLs which were associated with in-strain variability (isogenic discordance) in hepatic luciferase expression at week 3 PVA and with the ratio of hepatic to spleen vector copy number (VCN). These findings suggest that isogenic discordance regarding host permissiveness to the completion of different steps in the viral vector transduction process is genetically regulated and not uncommon.

In total, our results highlight the impact of genetic variation on initial responses, biodistribution, and maintenance of lentivirus-based hepatic transgene delivery. The wide range of strain-specific permissiveness to hepatic lentiviral vector transduction suggests that a modified, more conservative approach should be considered in designing preclinical and clinical gene therapy protocols for non-fatal diseases.

## 2. Materials and Methods

### 2.1. Mice

The animal protocol (IACUC 15-270.0) and all procedures were approved by the Institutional Animal Care and Use Committee of UNC.

Collaborative Cross and C57BL6 mice were obtained from UNC Systems Genetics Core Facility (SGCF).

The 41 Collaborative Cross strains included the following: CC002/Unc, CC003/Unc, CC004/TauUnc, CC005/TauUnc, CC006/TauUnc, CC008/GeniUnc, CC010/GeniUnc, CC011/Unc, CC012/GeniUnc, CC013/GeniUnc, CC016/GeniUnc, CC019/TauUnc, CC021/Unc, CC024/GeniUnc, CC027/GeniUnc, CC030/GeniUnc, CC032/GeniUnc, CC033/GeniUnc, CC035/Unc, CC036/Unc, CC038/GeniUnc, CC040/TauUnc, CC041/TanUnc, CC042/GeniUnc, CC043/GeniUnc, CC044/Unc, CC046/Unc, CC053/Unc, CC055/TauUnc, CC056/GeniUnc, CC057/Unc, CC058/Unc, CC059/TauUnc, CC060/Unc, CC061/GeniUnc, CC063/Unc, CC065/Unc, CC068/TauUnc, CC070/TauUnc, CC072/TauUnc, and CC076/Unc (named below as CC###).

### 2.2. Lentiviral Particles Production, Concentration, and Titration

A lentiviral vector (pTK979) carrying the firefly luciferase cDNA under the control of a liver-specific promoter (human alpha1-antitrypsin, hAAT) was constructed and used as previously described [28]. VSV-G pseudotyped lentiviral vector particles were packaged using the packaging cassette ΔNRF in 293T cells using three-plasmid transient transfection, as previously described [29]. Vector titers were determined by measuring the p24 capsid concentration using p24 ELISA, as previously described [30]. All vector preps were tested for replication-competent retroviruses using three independent assays, as previously described [31].

### 2.3. Lentiviral Vector Administration and in Vivo Imagine

A total of 25 µg p24^gag^ VSV-G pseudotyped lentiviral vectors were intraperitoneally injected into each mouse. In vivo expression of vector-delivered firefly luciferase in live animals was determined at weeks 1, 3, 6, 8, 14, and 24 using the IVIS Lumina optical system (PerkinElmer, Waltham, MA, USA), as described previously [8].

### 2.4. Tissue Vector Copy Number

Genomic DNAs from the liver and spleen were isolated using the Purelink Genomic mini-DNA kit (Thermo Fisher Scientific, Waltham, MA, USA). An additional RNaseA (Fermentas, Waltham, MA, USA) digestion step was added during DNA extraction to remove excessive RNAs. The total viral copy number (VCN) was quantified by multiplex PCR on an ABI7300 Realtime PCR system, as described previously [32].

### 2.5. Sleep Phenotyping and Behavior Analysis

Mice of the C57BL/6 strains and the 011, 013, 027, 033, 036, 041, 057, 058, 060, and 072 CC strains were moved to our wake/sleep behavior satellite facility, which was maintained on a 12 h–12 h light–dark cycle (lights on 7 am to 7 pm). Individual mice were housed in 15.5 cm [2] cages with bedding, food, and water. Before the beginning of data collection, the mice were allowed to acclimate to the environment for at least two full dark cycles. No other animals were housed in the room during these experiments. Sleep and wake behavior were recorded using a noninvasive home-cage monitoring system, PiezoSleep 2.0 (Signal Solutions, Lexington, KY, USA), as previously described [33]. Briefly, the system uses a piezoelectric polymer film to quantitatively assess sleep/wake cycles, total amount of sleep, and sleep quality from mechanical signals obtained from via breath rate and movement. Specialized software (SleepStats, Signal Solutions, Lexington, KY, USA) uses an algorithm to discern sleeping respiratory patterns from waking respiratory patterns. Sleep was characterized according to specific parameters, in accordance with the typical respiration of a sleeping mouse. Additional parameters were set to identify wake states, including the absence of characteristic sleep signals and higher amplitude, irregular signals associated with volitional movements, and even subtle head movements during quiet wake states. Data collected from the cage system were binned for every 1 h to generate a daily sleep trace or 12 h bins for average light- or dark-phase sleep, and light over dark ratios were calculated. This algorithm has been validated in adult mice using electroencephalography, electromyography, and visual evaluation [34,35,36,37] and has been successfully utilized in additional studies [38,39].

### 2.6. Statistical and QTL Analyses

We employed 254 female mice from C57B6 and 41 Collaborative Cross (CC) strains, with four to eight replicates per strain. We measured hepatic luciferase expression at weeks 1, 3, 6, 8, 14, and 24 as well as liver and spleen VCN at the end of the experiment. We calculated the difference in hepatic luciferase expression between different weeks, the ratio of this difference to the hepatic luciferase at the earlier week (e.g., (Luc expression at week 1 − Luc expression at week4)/Luc expression at week 1), and the vector-specific activity (SA) as the ratio of hepatic luciferase to liver VCN. All statistical analyses were conducted using R (version 4.2.1). To identify potential outliers, hepatic luciferase expression, VCN, and specific activity were first log transformed, and then their robust estimates of mean and standard deviation were calculated (per strain) using winsorized statistical measurements [40]. The ratio of difference to the hepatic luciferase expression at the previous week were added before log transformation, since some ratios were negative. Observations more than 4 standard deviations from the mean were flagged as outliers, resulting in 11 outliers across 3 phenotypes. All QTL analyses were performed on the cleaned data, with the outliers being removed.

To estimate the broad-sense heritability of each phenotype, we performed a one-way ANOVA, where the CC strains were modeled as a categorical variable. Broad-sense heritability was defined as the percentage of the phenotype variance explained by the CC strains. 

We downloaded the probability matrix of 36 genotype calls (8 homozygous calls and 28 heterozygous calls) across 76,689 SNPs from https://csbio.unc.edu/CCstatus/index.py?run=FounderProbs (accessed on 26 January 2021), which were then converted into an 8-state allele probability matrix. We performed QTL mapping at each SNP using the qtl2 package in R. The genome-wide significance was empirically assessed through 1000 permutations. Phenotypes with top candidate loci that passed the genome-wide significance threshold of 0.2 are listed in the results section, with their corresponding *p*-value, LOD scores, Bayes credible intervals, and the heritability of the top SNP.

## 3. Results

### 3.1. Overall Approach

This study is based on the notion that long-term expression of a therapeutic level of lentiviral vector-delivered genetic cargos, without adverse effects, is the hallmark of efficacious therapeutic gene therapy protocols. However, variations in the outcomes of viral vector-based gene therapy among patients bounds the usage of gene therapy protocols to incurable human diseases [9,10,11,12,13,14]. We assume that the phenomenon of patient-specific outcomes is secondary to the host genetic background. We believe that identification of specific host genetic loci associated with the efficacy and safety of lentiviral vector gene delivery would improve our ability to predict the outcomes of therapeutic gene delivery and would pave the way for the development of personalized gene therapy protocols. Our approach of achieving this goal is premised on associating quantifiable phenotypes of lentiviral vector-mediated gene delivery with the genetic background of 41 genetically diverse CC mouse strains. This strategy was supported by an earlier pilot study demonstrating strong host genetic effects on the efficacy of lentiviral vector hepatic gene delivery in 12 CC mouse strains [8]. The vector copy number (VCN) in target organs and vector-specific activity (transgene expression level per vector genome) determine the long-term transgene expression level. Two intrinsic host mechanisms negatively affect long-term transgene expression from lentiviral vectors. Epigenetic silencing of the transgene expression cassette would result in an overall reduction in transgene expression, without affecting the VCN, while transgene induced cytotoxicity or cellular immune response directed to vector-transduced cells would reduce VCN and overall transgene expression levels. Note that relatively rapid uncoating of HIV-1-based vectors [41] and the lack of earlier exposure of CC mice to HIV antigens render the possibility that the cellular immune response to HIV-1 antigens will contribute to a decline in transgene expression in vector transduced mice less likely. However, it is not completely improbable [42]. Accordingly, the study presented here aimed at identifying host genetic loci associated with the above parameters that affect the long-term outcomes of lentiviral vector transduction. The study was divided into two parts. The first part comprised data collection, in which we periodically quantified hepatic transduction efficiency and analyzed more than 23 traits of lentiviral vector-mediated hepatic gene delivery in multiple CC mouse strains. In the second part of this study, based on these findings and on the well-characterized genetic background of CC mouse strains, we identified quantitative trait loci (QTLs) that were linked by association to strain-specific characteristics of hepatic gene delivery by lentiviral vectors.

### 3.2. Broad Range of Lentiviral Vector-Mediated Hepatic Luciferase Expression Among 41 Collaborative Cross Mouse Strains

**Figure 1:**
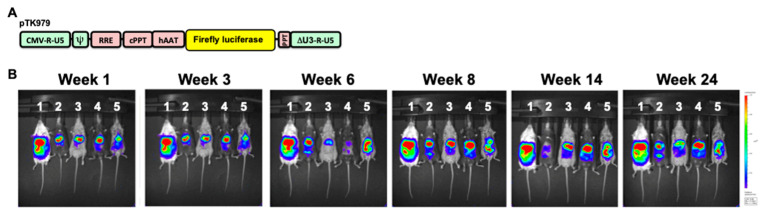
Hepatic transduction by lentiviral vectors in Collaborative Cross (CC) mice. (**A**) Depiction of the lentiviral vector pTK979, from which the firefly luciferase is expressed under the control of the liver-specific promoter hAAT. The vector’s 5′ and 3′ LTRs, packaging signal (y) (Ψ), rev response element (RRE), and the central and 3′ polypurine tract (cPPT and PPT, respectively) are shown. (**B**) In vivo imaging of firefly luciferase expression in a sample of five CC mice at weeks 1, 3, 6, 8, 14, and 24 days post vector administration (PVA) following intraperitoneal injection with lentiviral vectors expressing the firefly luciferase under the control of a liver-specific promoter (hAAT). The mice were imaged using the IVIS in vivo imaging system.

**Figure 2:**
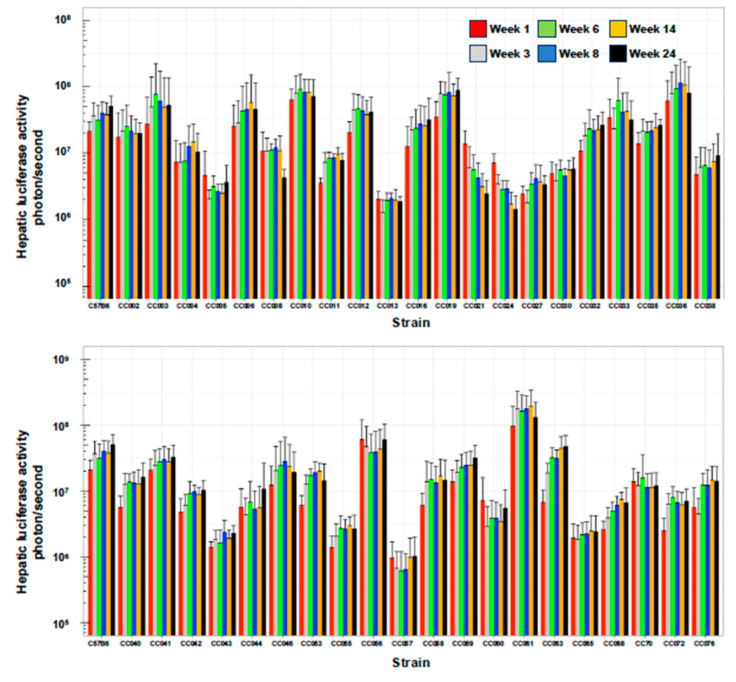
The kinetics of hepatic transgene (firefly luciferase) expression following intraperitoneal administration of lentiviral vectors. Bar graph showing levels of hepatic luciferase expression in C57B6 and 41 CC mouse strains between weeks 1 and 24 PVA. To characterize the effect of host genetic variations on hepatic gene delivery by lentiviral vector female mice from C57B6 and 41 CC mouse strains were intraperitoneally injected with VSV-G pseudotyped lentiviral vectors (pTK979). Luciferase expression in mouse livers was periodically determined at 1, 3, 6, 8, 14, and 24 weeks PVA using the IVIS imagine system.

**Figure 3:**
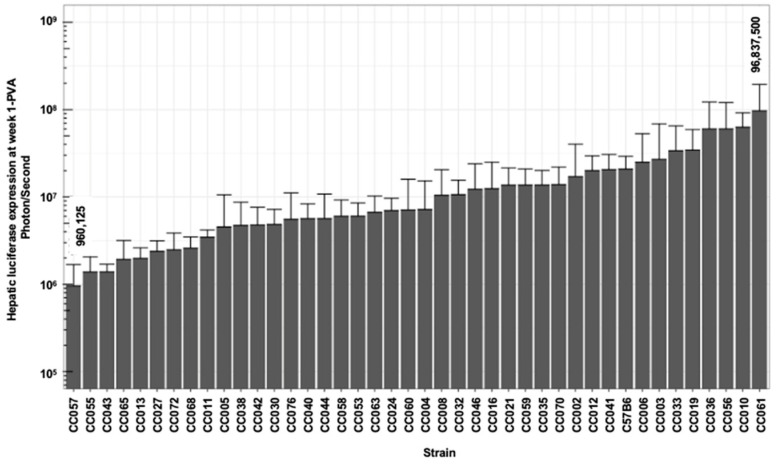
Hepatic luciferase expression at week 1 PVA in CC mouse strains. Bar graph showing the mean hepatic luciferase expression (photon/second) at week 1 PVA. The identity of the relevant CC mouse strains is shown in the bottom of the graph. The level of the lowest and highest means of hepatic luciferase expression (960,125 photons/second in CC057 and 96,837,500 in CC061, respectively) are shown.

**Figure 4:**
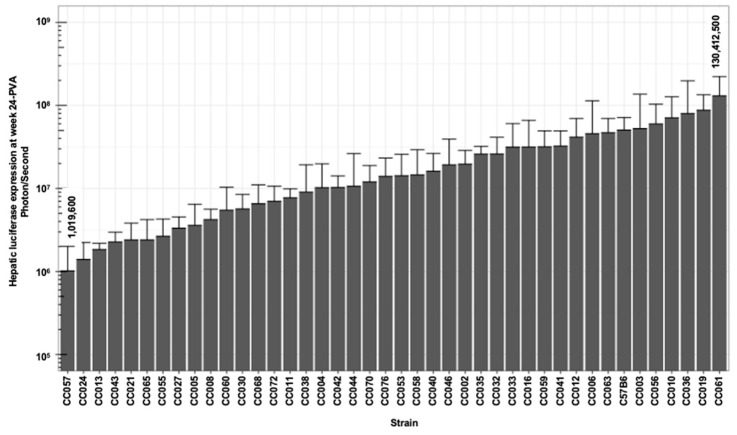
Hepatic luciferase expression at week 24 PVA in CC mouse strains. Bar graph showing the mean hepatic luciferase expression (photon/second) at week 24 PVA. The identity of the relevant CC mouse strains is shown in the bottom of the graph. The level of the lowest and highest means of hepatic luciferase expression (1,016,900 photons/second in CC057 and 130,412,500 in CC061, respectively) are shown.

We sought to characterize strain-specific traits affecting the abovementioned aspects of lentiviral vector mediated hepatic gene delivery. To this end, VSV-G pseudotyped lentiviral vectors carrying the firefly luciferase cDNA under the control of a liver-specific promoter (hAAT) were intraperitoneally administered to 254 females of the C57B6 and 41 CC mouse strains. Hepatic luciferase expression was quantified periodically for 24 weeks (Figure 1, Figure 2, Figure 3 and Figure 4).

As shown in Figure 2, Figure 3 and Figure 4 and Table 1, we observed a broad range (up to a 200-fold difference) of hepatic transgene (firefly luciferase) expression levels among the CC mouse strains. The means of hepatic luciferase expression among all CC mouse strains at week 1 (first measurement) and week 24 (last measurement) were 1.61 × 10^7^ and 2.51 × 10^7^ photon/s, respectively. Strains CC061 and CC057 exhibited the highest and lowest means of hepatic luciferase expression at week 1 and week 24 PVA (9.68 × 10^7^ and 9.60 × 10^5^ photon/s at week 1 and 1.30 × 10^8^ and 1.02 × 10^6^ photon/s at week 24 PVA, respectively).

As shown in Figure 2, Figure 3 and Figure 4, the means of the luciferase expression levels at week 24 PVA in 15 of the 42 studied strains (35.7%) were lower than 10^7^ photons/second. In an additional 25 mouse strains (59.5%), the means of the luciferase expression levels at week 24 PVA were higher than 10^7^ photons/second. These data underscore the high variability in hepatic gene delivery by the lentiviral vector among the CC mouse strains.

### 3.3. Hepatic and Splenic Lentiviral Vector Genome Copy Number per Cell (VCN) in CC Mouse Strains

**Figure 5:**
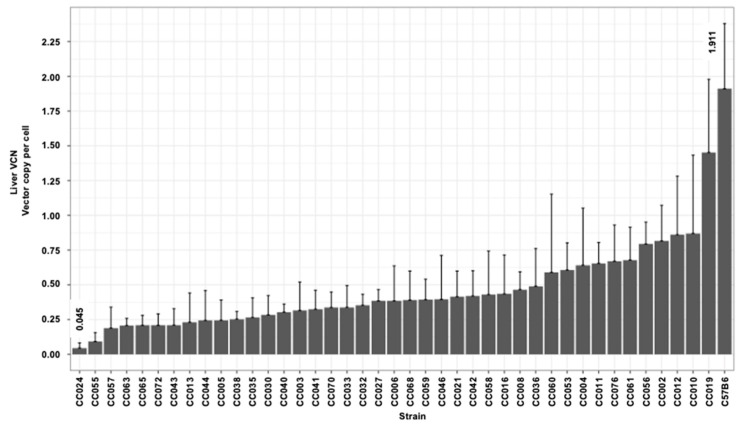
Liver VCN in CC mouse strains. Bar graph showing means of VCN in liver tissues from 41 CC mouse strains, as determined by qPCR. The highest and lowest liver VCN (1.911 in C57B6 and 0.045 in CC024, respectively) are shown.

**Figure 6:**
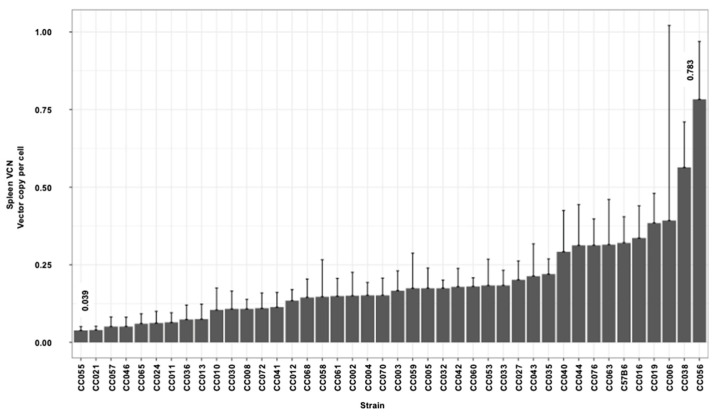
Spleen VCN in CC mouse strains. Bar graph showing means of VCN in liver tissues from 41 CC mouse strains, as determined by qPCR. The highest and lowest liver VCN (0.783 in CC056 and 0.039 in CC055, respectively) are shown.

**Figure 7:**
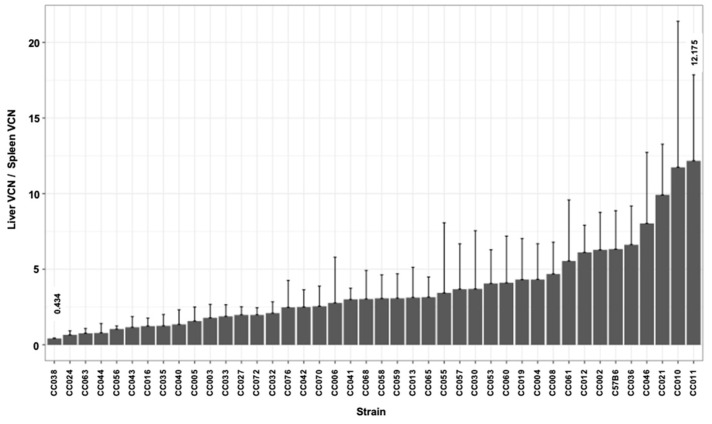
The Ratio of liver VCN to spleen VCN. Bar graph showing the means of liver VCN to spleen VCN ratios in lentiviral vector-treated CC mouse strains. VCN in the above tissues was determined by qPCR. The highest and lowest ratios in CC011 (12.175) and in CC036 (0.434), respectively, are shown.

The delivery of reverse-transcribed lentiviral genomes to the nuclei of the target cells (hepatocytes) culminates a multistep process. The efficiency of each of these steps directly affects the overall outcome of hepatic gene delivery. The VCN in transduced mouse tissues is a surrogate marker of lentiviral vector gene delivery efficiency. To further characterize the effects of the host genetic background on lentiviral vector-mediated hepatic gene delivery, we employed qPCR-analysis to determine the hepatic and splenic VCN in all vector transduced mice. As shown in Figure 5 and Table 1, we observed a greater than 40-fold difference between the highest and the lowest means of the hepatic VCN in C57B6 (highest VCN 1.911) and CC024 (lowest VCN 0.045), respectively. The mean hepatic VCN of all studied mouse strains was 0.47 vector genome per cell. The overall mean VCN in all splenic tissues of all mouse strains (0.19 vector genome per cell) was lower than that observed in the hepatic tissues (Figure 6 and Table 1). The lowest and highest means of splenic VCN (0.04 and 0.78, respectively) were exhibited by strains CC055 and CC056, respectively. Note that these mouse strains (CC055 and CC056) are different from their counterparts that exhibited the lowest and highest hepatic VCN (CC024 and C57B6, respectively). To better understand the mechanisms affecting the VCN in mouse tissues, we determined the ratio of hepatic to splenic VCN in all studied mice. As shown in Figure 7 and Table 1, the lowest (0.43) and highest (12.18) mean of hepatic VCN to splenic VCN ratio (in strains CC038 and CC011, respectively) differed by more than 28-fold. Altogether, these data suggest that the level of host factors affecting the VCN of the CC strains are both strain- and cell type-specific.

**Figure 8:**
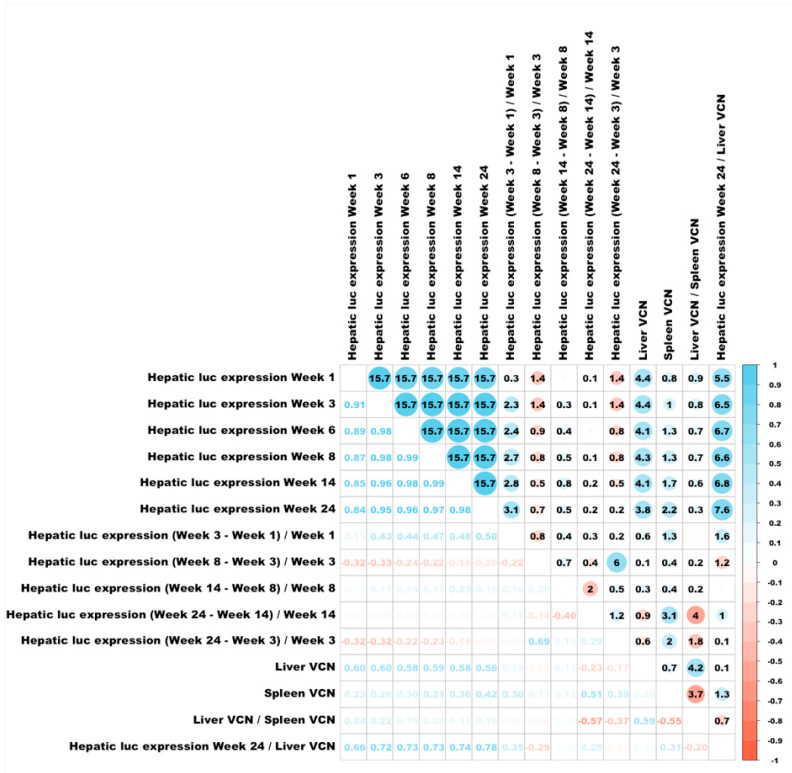
A visualization of the Spearman correlation matrix of hepatic luciferase expression and VCN. For the upper triangular correlation matrix, the areas of circles show the absolute value of corresponding correlation coefficients, and the numbers are −log10 of the *p*-value of tests for correlation. The positive correlations are shown in blue, and negative correlations are shown in red. The lower triangular correlation matrix shows coefficients numbers in different colors.

As expected, we found good correlation between the means of the lentiviral vector transgene expression and the mean of the hepatic VCN (Figure 8).

### 3.4. The Kinetics of Hepatic Luciferase Expression Level in CC Mouse Strains

**Figure 9:**
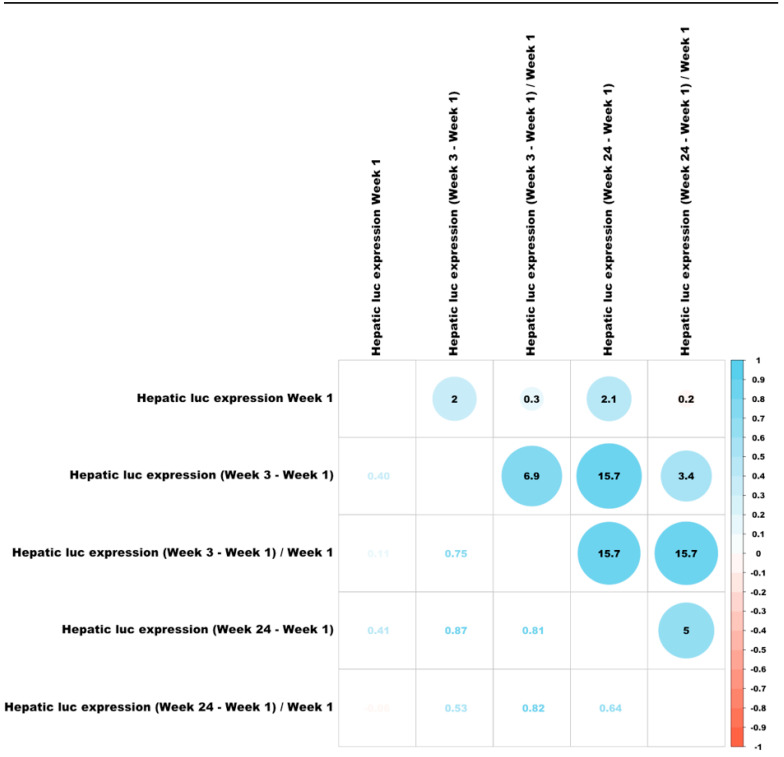
A visualization of the Spearman correlation matrix of hepatic luciferase expression at week 1 PVA and the differences in luciferase expression levels between week 1 PVA and weeks 1 and 24 PVA. For the upper triangular correlation matrix, the areas of circles show the absolute value of corresponding correlation coefficients, and the numbers are -log10 of the *p*-value of tests for correlation. The positive correlations are shown in blue, and negative correlations are shown in red. The lower triangular correlation matrix shows coefficients numbers in different colors.

**Figure 10:**
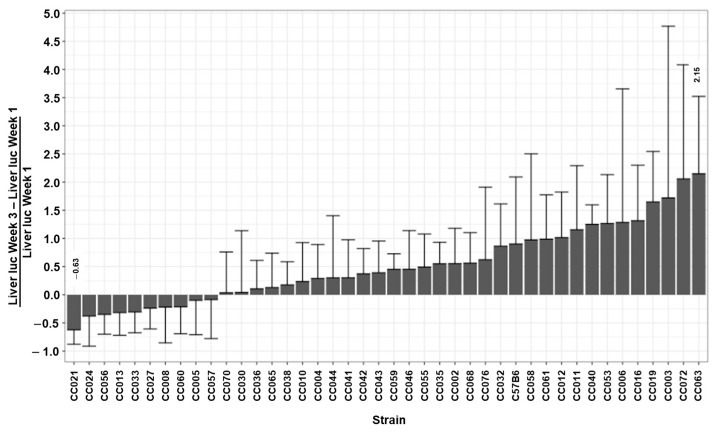
The kinetics of hepatic transgene expression between week 1 and week 3 PVA. A bar graph showing the difference in the mean hepatic transgene expression between week 1 PVA and week 3 PVA in the abovementioned 41 CC and C57B6 mouse strains. To minimize the intrinsic effect of strain-specific transgene expression at week 1 PVA on the magnitude of the difference in transgene expression at weeks 1 and 3 PVA, the calculated differences were divided by the value of transgene expression at week 1 PVA. The highest and lowest means (2.15 in CC063 and −063 in CC021, respectively) are shown.

**Figure 11:**
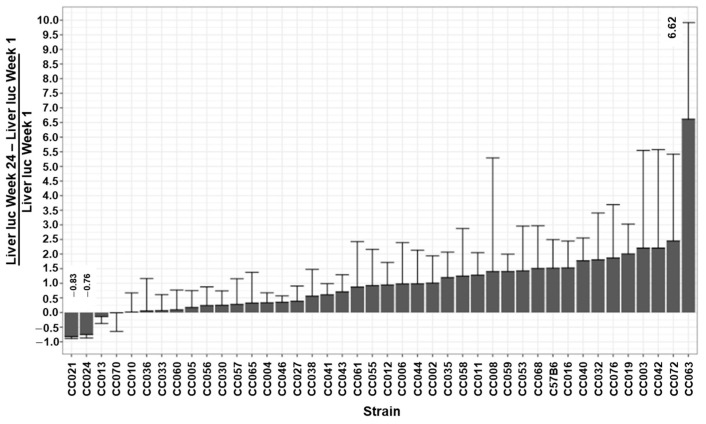
The kinetics of hepatic transgene expression between week 1 and week 24 PVA. A bar graph showing the difference in the mean hepatic transgene expression between week 1 PVA and week 24 PVA in the abovementioned 41 CC and C57B6 mouse strains. To minimize the intrinsic effect of strain-specific transgene expression at week 1 PVA on the magnitude of the difference in transgene expression at weeks 1 and 24 PVA, the calculated differences were divided by the value of transgene expression at week 1 PVA. The highest and lowest means (6.62 in CC063 and −0.83 in CC021, respectively) are shown.

The ability to maintain therapeutic levels of transgene expression is the hallmark of all successful gene replacement therapies. To study the effects of the host genetic background on the kinetics of hepatic transgene expression from lentiviral vectors, we determined the differences between hepatic luciferase expression levels at week 1 PVA and luciferase expression levels at weeks 3 and 24 PVA (D of W3-1 and D of W24-1, respectively). Since the levels of luciferase expression at week 1 PVA intrinsically affect the absolute value of the above Ds (Figure 9), we normalized the effects of W1 PVA expression levels by dividing the values of the above Ds by the values of their respective expression levels at W1 PVA (∆W3-1/W1 and ∆W24-W1/W1). As shown in Figure 2, Figure 10, and Figure 11 and Table 1, C57B6 mice, and most of the CC mouse strains, exhibited a stable or an increased hepatic luciferase expression throughout the study. However, two mouse strains, CC021 and CC024, showed a significant decrease in hepatic transgene expression levels between Weeks 1 and 24 PVA. Specifically, W24-W1/W1 results of −0.81 and −0.76 were expressed by CC021 and CC024, respectively. This phenomenon could have been mediated by either specific elimination of luciferase-expressing hepatocytes and/or by transcriptional silencing of the lentiviral vector expression cassettes. We theorized that VCN in hepatic and splenic tissues in CC021 and CC024 mouse strains could imply a role of the above two mechanisms in the decrease in hepatic transgene expression from the lentiviral vectors. As shown in Figure 5 and Figure 7, hepatic tissues in CC024 mice exhibited the lowest levels of VCN, and the second lowest hepatic VCN to splenic VCN ratio. These findings support the hypothesis that the elimination of luciferase-expressing hepatocytes probably contributed to the decrease in hepatic transgene expression from the lentiviral vectors. Per contra, CC021 mice exhibited moderate levels of hepatic VCN and the third highest ratio of hepatic to splenic VCN. Based on these data, we suggest that transcriptional silencing of the lentiviral vector cassettes contributed to the decrease in hepatic luciferase expression between weeks 1 and 24 PVA in CC021 mice.

### 3.5. Lentiviral Vector-Specific Activity in CC Mouse Strains

**Figure 12:**
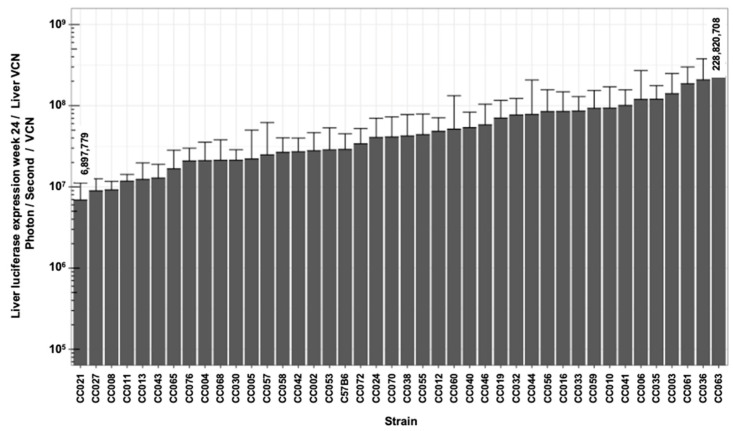
Specific activity of lentiviral vectors carrying a liver-specific promoter in CC mouse strains. A bar graph showing means of vector-specific activity in C57B6 and 41 CC mouse strains. To determine vector-specific activity, hepatic luciferase activity at week 24 PVA was divided by liver VCN. The highest and lowest means of vector-specific activity (228,820,708 photon/s/vector copy per cell, in CC063 and 6,897,779 photon/s/vector copy per cell in CC021, respectively) are shown.

The above findings implied that the strain-specific transcriptional regulation of lentiviral vector cassettes controls hepatic transgene expression. Thus, we sought to characterize the specific activity (SA) of the lentiviral vectors carrying a liver-specific promoter (hAAT) in the above panel of CC mouse strains. To this end, the vector SA activity in each of the studied mice was calculated by dividing the mouse hepatic luciferase expression level at week 24 PVA by the mouse hepatic VCN. The CC mouse panel exhibited a wide range of mean lentiviral vector SA (Figure 12 and Table 1). Specifically, a greater than 30-fold difference in vector SA was observed between the lowest and highest means of vector SA (6.9 × 10^6^ photons/s/VCN in mouse strain CC021 and 2.3 × 10^8^ photons/s/VCN in mouse strain CC063, respectively). These findings indicate that in addition to VCN, vector-specific activity, which is determined by the host transcription factor profile, and the epigenetics of the vector affect the outcomes of gene delivery in a strain-specific manner.

### 3.6. Correlations Between Different Characteristics of Hepatic Transduction by Lentiviral Vectors

To better understand the mechanisms affecting hepatic transduction efficiency by lentiviral vectors, we calculated the correlation between the above traits (Figure 8). Hepatic luciferase expression levels in weeks 1- to 14 PVA highly correlated with hepatic luciferase expression at week 24 PVA. A mild increase in Spearman correlation was observed between luciferase expression at weeks 1 and 24 PVA (0.84) and weeks 3 and 24 PVA (0.95). As expected, the levels of hepatic VCN positively correlated with hepatic transgene expression throughout the study (R ≥ 0.56). Vector SA (hepatic luciferase expression at week 24 PVA/VCN) positively correlated with hepatic luciferase expression at weeks 1 to 14 PVA. The lowest level of Spearman correlation of vector SA was observed with week 1 PVA (0.66). A mild increase in Spearman correlation was observed at week 3 PVA (0.72) and was comparable to the Spearman correlation at weeks 6, 8 and 14 PVA). Except for Spearman correlation between hepatic luciferase expression at week 24 PVA and the differences observed in hepatic luciferase expression at weeks 1 and 3 PVA (0.50), differences in hepatic luciferases expression between the different time points during the study did not correlate with hepatic luciferase expression at week 24 PVA. Altogether, these findings raise the possibility that changes in hepatic luciferase expression level and vector SA between weeks 1 and 3 PVA have major effect on long-term hepatic transgene expression from lentiviral vectors.

### 3.7. In-Strain Variabilities in Characteristics of Hepatic Transgene Expression

**Figure 13:**
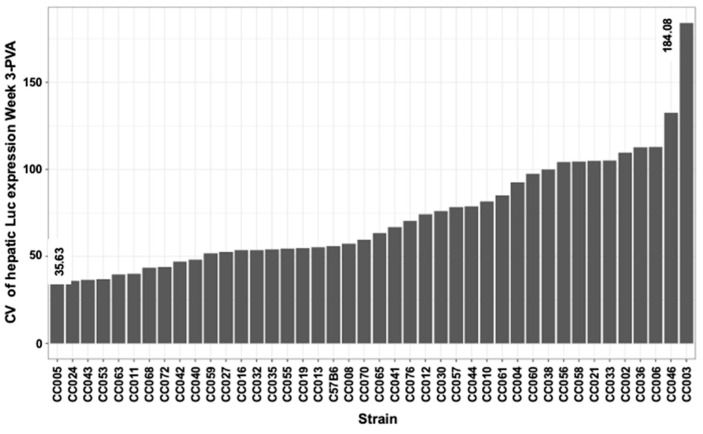
Coefficient variation (CV) of hepatic luciferase expression at week 3 PVA. Bar graph showing mouse strain-specific intrastrain variation in luciferase expression at week 3 PVA. Since mean luciferase expression levels intrinsically affect standard deviation (std) of luciferase expression. Coefficient variation (std/mean) was employed to characterize strain-specific effects on intrastrain variation in luciferase expression at week 3 PVA. The highest and lowest CV (184.08 in strain CC003 and 35.63 in CC005, respectively) are shown.

**Figure 14:**
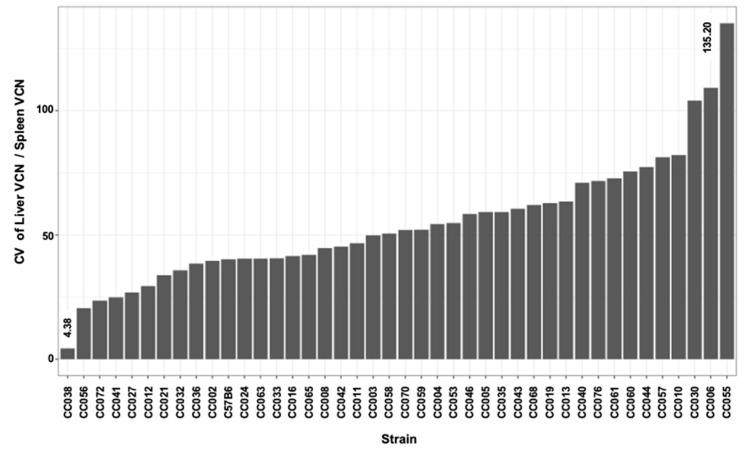
CV of liver VCN and spleen VCN ratios (liver VCN/Spleen VCN) in CC mouse strains. Bar graph showing mouse strain-specific intrastrain variation in liver VCN/spleen VCN ratios. Since the mean of the above ratios intrinsically affects the std of the above calculated ratios within each mouse strain, coefficient variation (std/mean) was employed to characterize strain-specific effects on intrastrain variation in liver VCN/Spleen VCN ratios. The highest and lowest CV (135.20 in strain CC055 and 4.38 in CC038, respectively) are shown.

**Figure 15:**
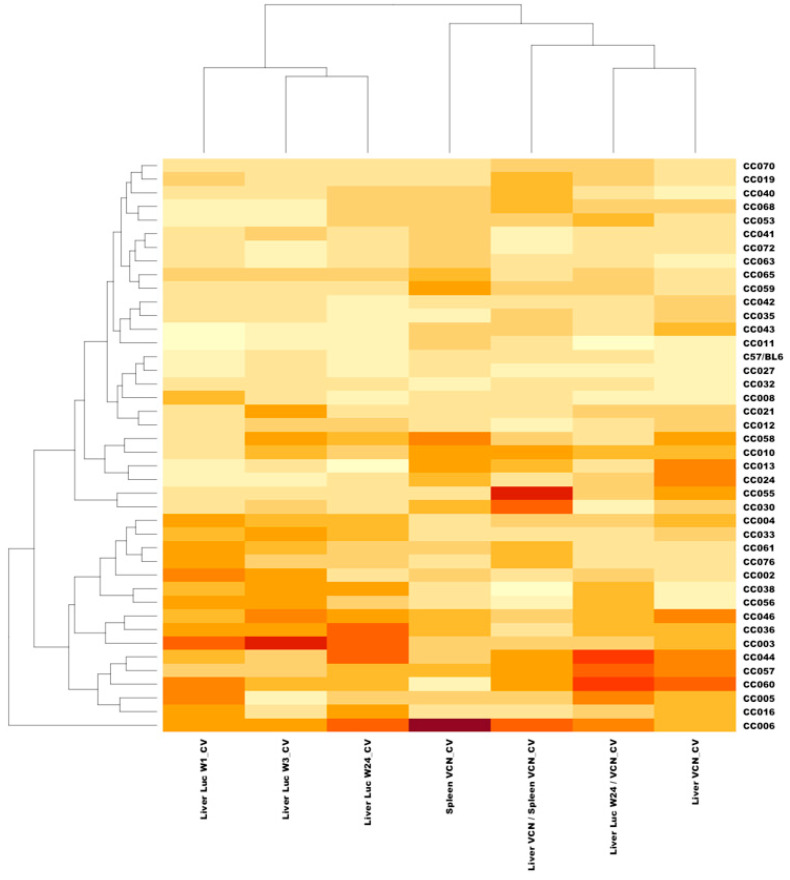
Heatmap of variables related to CV across mouse strains. Values are displayed as colors ranging from light yellow to dark red, and larger values are in darker colors. Both rows and columns are clustered using correlation distance with a dendrogram added to the left side and to the top.

Intrigued by the wide range of phenotypes, amongst the different CC mouse strains, we sought to characterize intrastrain variabilities in the above studied traits. To this end, coefficient variation (CV) of the above traits was calculated (CV = standard deviation/mean) for each CC mouse strain. As shown in Figure 13 and Figure 14 and Table 2, we observed a wide range of CVs of the studied phenotypes within the different CC mouse strains. These findings indicated that the host genetic background affects both strain and intrastrain-specific phenotypes and raised the possibility that specific host factors contribute to overall intrastrain phenotypic variability. Specifically, we sought to investigate if an increased CV in a specific phenotype increases the likelihood of observing an increase in CV of other phenotypes. As shown in a heatmap representation (Figure 15) specific mouse strains exhibited increased CV in several phenotypes some of which were not directly related. These findings suggest that the host genetic background could contribute to the overall genotypic phenotypic discordance in a trait specific manner and potentially the ability to respond to environmental pressures.

### 3.8. The Overall Effect of the Host Genetics on Hepatic Transduction by Lentiviral Vectors

To quantify the contribution of the host genetics to the characteristics of hepatic transduction by lentiviral vector in the CC mouse strain panel, we calculated global heritability of each phenotype that had replicates within each CC mouse strain. The corresponding *p*-values in testing the CC genetic effects and the global heritability are summarized in Table 3. As shown, heritability of most phenotypes (12 out of 16) ranged between 43.19% to 59.02% with *p*-values < 0.0001. Heritability of four phenotypes which focused on the kinetics of hepatic transgene expression after week 3 PVA was low (14.79–23.01%) with *p*-values 0.613–0.037. Per contra the global heritability of changes in hepatic luciferase expression between weeks 1 and 3 PVA was highly significant (*p*-value < 0.0001) and thus, further support the notion that changes in hepatic transgene expression from lentiviral vectors are likely to occur during the first three weeks post transduction. Overall, these data confirmed our notion that the host genetic background has a major role in establishing strain-specific characteristics of lentiviral vectors transduction. However, it appeared that non-genetic factors also contributed to the above studied phenotypes.

### 3.9. Mouse Strain-Specific Sleep Patterns as an Intrinsic Environmental Factor Affecting Hepatic Transduction by Lentiviral Vectors

**Figure 16:**
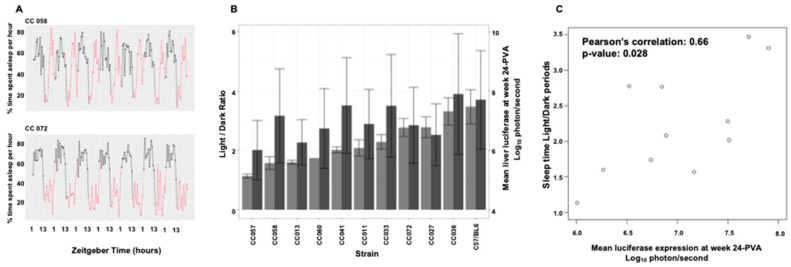
The effects of sleep patterns on hepatic transduction via lentiviral vectors. (**A**) The average percentage of time spent asleep per hour in 7-day sleep traces for CC058 and CC072. The x-axis represents zeitgeber time in hours. Black dots indicate lights on, and red dots indicate lights off. (**B**) Bar plots show the average ratio of time spent asleep during light periods compared to dark periods (light grey) and the log10 of mean liver luciferase at week 24 (dark grey) for each Collaborative Cross mouse strain. The error bars represent one standard deviation, with half a standard deviation above and below the mean. We calculated the average percentage of time spent asleep during light or dark periods for each mouse, then computed the ratio of time spent during light to dark periods, and finally, averaged ratios across all mice for each strain. (**C**) Scatter plots show the relationship between the average ratio of time spent asleep during light periods compared to dark periods and the log10 of mean liver luciferase at week 24; Pearson’s correlation = 0.66 (*p*-value = 0.028). There are 5 female mice in C57/BL6, 5 in CC011, 5 in CC013, 4 in CC027, 5 in CC033, 4 in CC036, 4 in CC041, 5 in CC057, 5 in CC058, 1 in CC060, and 4 in CC072, respectively, when calculating the ratio of time spent asleep during light periods compared to dark periods. There are 8 female mice in C57/BL6, 5 in CC011, 6 in CC013, 7 in CC027, 6 in CC033, 8 in CC036, 6 in CC041, 8 in CC057, 6 in CC058, 6 in CC060, and 6 in CC072, respectively, when calculating the mean liver luciferase at week 24.

The above findings suggested that in addition to the significant host genetic effects on hepatic lentiviral transduction, environmental factors probably contributed to the studied phenotypes. Importantly, in this study, all in vivo experiments were carried under a tightly controlled environment. We theorized that mouse-strain-specific sleeping patterns could generate an intrinsic environment, which directly affects adult mouse gene expression. Note that daily animal care (e.g., bedding replacement, cage care) and treatments in most academic facilities takes place during the mouse daylight/sleep period, which can result in continuous sleep disturbances, whose magnitude of effects could be mouse strain-specific. Furthermore, there is a possibility that aberrant maternal sleep patterns induce life-long behavioral and physiological alterations. To study the potential effects of sleep patterns on hepatic lentiviral vector transduction, we made use of sleep/wake behavior patterns, which were analyzed in a separate earlier experiment busing PiezoSleep in 11 random mouse strains (C57B6 and 10 CC mouse strains). The PiezoSleep system is an automated, non-invasive home-cage monitoring system that uses vibrational sensors to measure mouse motion and breathing to score wake/sleep behavior [34,35,38,39]. Mice are nocturnal; thus, it is expected that mice will display most of their sleeping behavior during the light period (Figure 16A). The percent of sleep time was determined hourly for 7 days in the above mouse strains (Figure 16A,B). To quantify sleep pattern behaviors, the averages of the ratios of percent sleeping time during light (zeitgeber 1–12) and dark (zeitgeber 13–24) periods were calculated, and these demonstrated a significant strain-specific phenotype (Figure 16B). Next, we calculated the correlation between the light/dark sleep ratios in the above 11 mouse strains and hepatic luciferase expression at week 24 PVA, which was determined in the respective mouse strains in the current study. As shown in Figure 16C, a moderate, yet significant, correlation (*p*-value = 0.028, and Pearson’s correlation = 0.66) was observed between the levels of hepatic transgene expression and the ratio of female mouse sleeping time during light and dark periods. Although highly intriguing, these findings should be further supported by using a larger number of CC mouse strains.

### 3.10. Identification and Characterization of QTLs Associated with Traits of Hepatic Lentiviral Vector Transduction

To better understand the role of the host genetic background in the process of hepatic transduction by lentiviral vectors, we sought to identify the genomic loci responsible for transcribing either protein encoding or non-protein coding RNAs that affect the above studied phenotypes. For this purpose, we performed a quantitative trait locus (QTL) analysis using the qtl2 package in R. Specifically, the significance of the linkage between genetic markers (SNPs) and the quantitatively characterized lentiviral vector hepatic transduction traits was determined by the LOD score (log10 of odds). This method measures the ratio between the probability of associating strain-specific SNPs with a linkage to the studied trait to the probability of associating strain-specific SNPs in the absence of linkage. The genome-wide significance was estimated based on the result from 1000 permutations.

Three trait-associated QTLs with *p*-values < 0.05 were identified (Table 4). The above traits include the coefficient variation (CV) of the ratio between liver VCN to spleen VCN, the CV of hepatic luciferase expression at week 3 PVA, and the difference between luciferase expression at week 3 and 1 PVA divided (normalized) by luciferase expression at week 1 PVA.

### 3.11. QTL Association with CV of the Ratio Between Liver VCN to Spleen VCN

**Figure 17:**
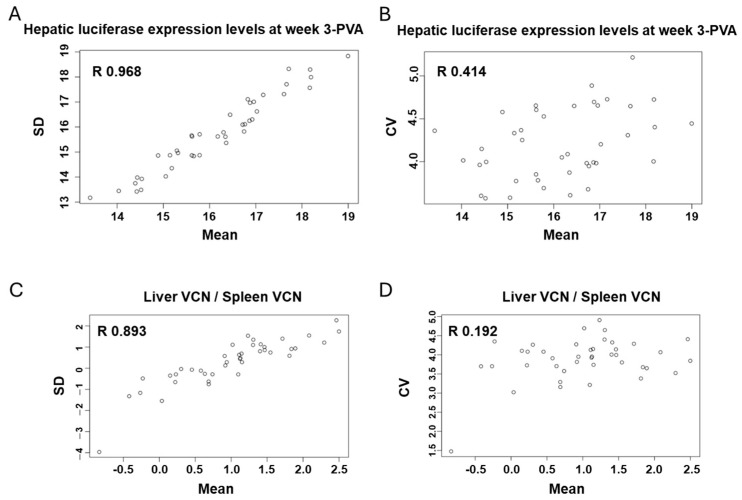
The intrinsic effects of mean luciferase expression levels on standard deviation (SD) and coefficient of variation (CV). (**A**) A scatter plot shows the relationship between the SDs and means of hepatic luciferase expression at week 3. (**B**) A scatter plot shows the relationship between the CVs and means of hepatic luciferase expression at week 3. (**C**) A scatter plot shows the relationship between the SDs and means of the ratio of liver and spleen VCN. (**D**) A scatter plot shows the relationship between the CVs and means of the ratio of liver and spleen VCN. The corresponding correlation coefficients are added. The correlation between the SDs and means of hepatic luciferase expression at week 3 is high, and the dots fall along the line.

**Figure 18:**
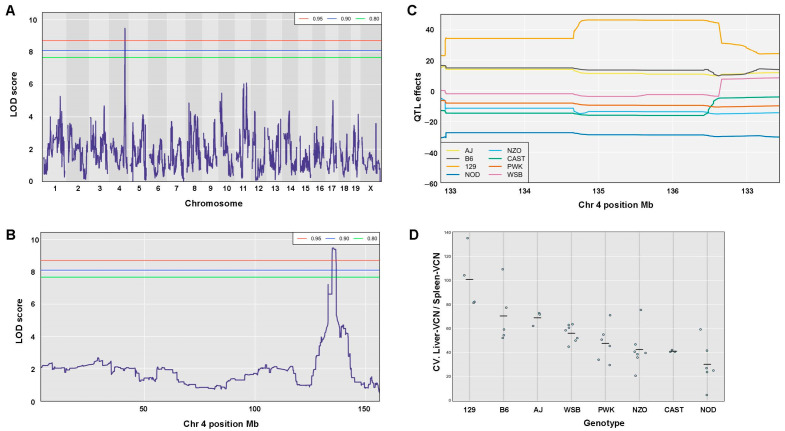
Host genetic background effects on intrastrain variation in the ratio of liver and spleen VCN. The CV (SD/mean) of the ratios of liver and spleen VCN in each CC mouse strain was employed to identify genomic loci associated with intrastrain variation in the above phenotype. (**A**) The LOD scores across all chromosomes. The horizontal red, blue, and green lines indicate the 5%, 10%, and 20% genome-wide significance threshold, respectively (based on a permutation test). (**B**) The LOD score for chromosome 4. (**C**) The estimated QTL effects of eight haplotypes among markers near the peak of QTL. (**D**) Dot plot showing the CV of the ratio of liver and spleen VCN in CC mouse stains carrying one of the eight founder alleles at the above QTL on chromosome 4. Note that four mouse strains carrying the 129s1/SvImJ haplotype exhibit the highest CVs.

**Figure 19:**
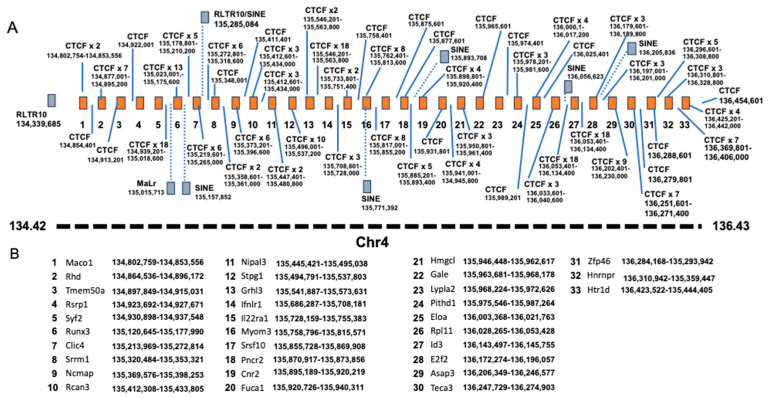
A physical map of a QTL in mouse chromosome 4, which associates with CVs in the ratios of liver and spleen VCNs. (**A**) Depiction of the above QTL located between Mbs 134.42 and 136.43 in chromosome 4. Numbered red boxes show the genes comprising the above QTL. Blue boxes show the various transposable elements and their locations in chromosome 4 (determined by Nellaker et al., 2012 (43) and converted to Ensmbel GRCm38.6 using https://genome.ucsc.edu/cgi-bin/hgLiftOver, accessed on 5 December 2024). CTCF target sequences and their location in chromosome 4 are shown (determined by Ensmbel GRCm38.6). (**B**) Names and locations of the respective numbered red boxes shown in (**A**).

The phenomenon of strain-dependent intrastrain variations in traits of hepatic lentiviral vector transduction (Figure 13 and Figure 14 and Table 2) raised the possibility that this phenomenon is affected by the host genetic background. This concept suggests that the host genetic background determines the magnitude of the isogenic phenotypic discordance of specific traits and potentially, the contribution of environmental effects on this phenomenon. To test this hypothesis and to identify QTLs associated with intrastrain variations, the CVs of the hepatic lentiviral vector transduction phenotypes were treated as traits. This approach was premised on the notion that in contrast to standard deviations (SDs), CVs are less affected by the size of the respective means. Thus, the likelihood of identifying genetic loci, which directly affect a CV of a trait, is higher compared to those that affect SD. Indeed, as shown in Figure 17, both the means of strain luciferase expression at week 3 PVA and the means of the ratios of liver VCN to spleen VCN correlated better with the strain SDs (R = 0.968 and 0.893, respectively) than with the strain CVs (R = 0.414 and 0.192, respectively). As shown in Figure 18A–D and Table 4, QTL analysis of the CV of the ratios of liver VCN means to spleen VCN means, identified a single QTL in chromosome 4, with a LOD score of 9.47 and *p*-value of 0.008. The QTL is positioned in Mb 135.41, and its width is 1.61 Mb. It comprises 33 protein coding genes and 11 pseudogenes. The percent of contribution (heritability) from the top SNP in the QTL regions to the above phenotype is 65.48%. As shown in Figure 18C,D, 4 CC mouse strains (CC010, CC030, CC055, CC057), which contain the 129s1/SvImJ allele in the above QTL, are associated with the highest intrastrain variation (CV) in the ratio of liver VCN to spleen VCN. Aware of the role of CCCTC-binding factor (CTCF) and retroelements in the phenomenon of isogenic phenotypic discordance and genomic imprinting [43,44,45,46], we mapped CTCF binding sequences in the QTL. As shown in Figure 19, 247 CTCF binding sites were identified in the above QTL. A total of 7 129s1/SvImJ-unique retroelements insertions were identified, including 5 SINES, an RLTR10, and a MaLr [45]. Furthermore, the location of the above MaLr insertion in the CTCF cluster raises the possibility that in line with earlier reports, interactions between the retroelements and the CTCF binding sequences may contribute to the increased CV of the above phenotype [43,44,45,46,47].

### 3.12. QTL Association with CV of Hepatic Luciferase Expression at Week 3 PVA

**Figure 20:**
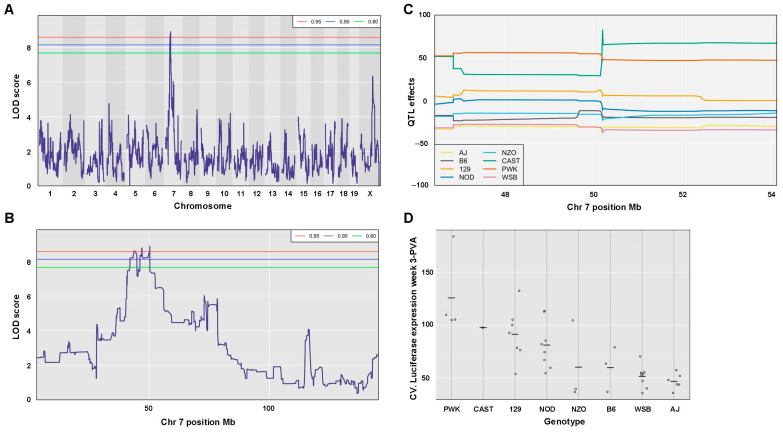
Host genetic background effects on intrastrain variation in hepatic luciferase expression at week 3 PVA. CV (SD/mean) of hepatic luciferase expression at week 3 PVA in each CC mouse strain was employed to identify genomic loci associated with intrastrain variation. (**A**) The LOD scores across all chromosomes. The horizontal red, blue, and green lines indicate the 5%, 10%, and 20% genome-wide significance threshold, respectively (based on a permutation test). (**B**) The LOD score of chromosome 7. (**C**) The estimated QTL effects of eight haplotypes among markers near the peak of QTL. (**D**) Dot plot showing the CV of hepatic luciferase expression at week 3 PVA in CC mouse stains carrying one of the eight founder alleles at the above QTL on chromosome 7. Note that four mouse strains carrying the PWK haplotype exhibit the highest CVs.

A second QTL with an LOD score of 8.92 and a *p*-value of 0.030 was associated with CV (strain-specific intrastrain variation) of hepatic luciferase expression levels at week 3 PVA (Figure 20A–D and Table 4). The QTL is positioned in 50.19 Mb of chromosome 7. Its width is 9.28 Mb, and it comprises 372 genes and 134 pseudogenes. The percentage of contribution (heritability) of the top SNP in the QTL region to the phenotype is 63.26%. As shown in Figure 20C,D and Table 4, CC mouse strains (CC002, CC003, CC033, CC058) which contain the PWK allele in the above QTL are associated with the highest CV.

### 3.13. Identification of a QTL Associated with Changes in Hepatic Luciferase Expression Between Weeks 1 and 3 PVA

**Figure 21:**
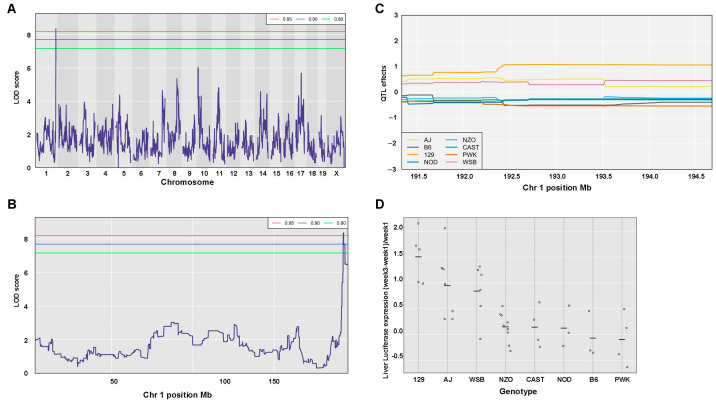
Characterization of host genetic background effects on the kinetics of hepatic luciferase expression between weeks 1 and 3 PVA. To this end, the differences in hepatic luciferase expression between weeks 1 and 3 PVA were normalized (divided) by the level of hepatic luciferase expression at week 1 PVA. (**A**) The LOD scores across all chromosomes. The horizontal red, blue, and green lines indicate the 5%, 10%, and 20% genome-wide significance threshold, respectively (based on a permutation test). (**B**) The LOD score for chromosome 1. (**C**) The estimated QTL effects of eight haplotypes among markers near the peak of the QTL. (**D**) Dot plot showing the means of normalized differences in hepatic luciferase between weeks 1 and 3 PVA in CC mouse strains carrying one of the eight founder haplotypes at the above QTL on chromosome 1. Note that five mouse strains carrying the 129s1/SvImJ haplotype exhibit the highest increase in hepatic luciferase expression between weeks 1 and 3 PVA.

The kinetics of hepatic transgene expression following the nuclear import of lentiviral vectors is one of the factors determining the outcomes of gene therapy protocols. To identify genomic loci, which are associated with changes in hepatic transgene expression from lentiviral vectors, differences between hepatic luciferase expression levels at weeks 1 and 3 PVA were determined. To minimize the intrinsic effects of transgene expression levels on the ability to associate the kinetics of transgene expression and specific QTLs, the above differences in expression levels at weeks 1 and 3 PVA were normalized by dividing the above differences in luciferase expression by the levels of luciferase expression at week 1 PVA. As shown in Figure 21A–D and Table 4, QTL analysis identified a single QTL, with an LOD score of 8.37 and a *p*-value of 0.04. It is associated with an increase in transgene expression between week 1 and week 3 PVA. The QTL is positioned at 192.36 Mb of chromosome 1. It encompasses 1.35 Mb, and the top SNP in the QTL region contributes 60.92% to the above phenotype. As shown in Figure 21C,D, five CC mouse strains (CC003, CC012, CC019, CC061, CC063) which are composed of the 129s1/SvImJ allele in the above QTL are associated with the highest increase in transgene expression between week 1 and week 3 PVA. Importantly, the above QTL interval comprises 34 genes and four pseudogenes, including five protein encoding sequences, which potentially affect the kinetics of transgene expression between week 1 and week 3 PVA. These genes include (A) *SERTAD4* (involved in cell cycle progression and chromatin remodeling) [48,49], (B) *IRF6* (a regulator of development and the innate immune response) [50,51,52,53], (C) *TRAF3IP* (a regulator of the innate immune response) [54,55], and (D) *UTP25/DIEFX* (a regulator of development, ribosome biogenesis, and tumor development) [56,57,58,59]. Importantly, a recent study [60] reported on chromatin-based interactions between genetic variants of the UTP25 promoter and the *TRAF3IP3* and *IRF6* genes in immune cells, which are involved in an autoimmune disorder.

## 4. Discussion

Empirically tailoring conventional therapeutic regimens to an individual patient only partially compensates for our limited ability to accurately predict the safety and efficacy of therapeutic protocols for an individual patient. Furthermore, this methodology cannot be applied to gene therapy protocols, which are premised on a single administration procedure.

Variability in the outcome of viral vector-based gene therapy clinical trials can be attributed to patient-specific expression profiles of viral dependence and viral restriction factors. An increasing number of host factors have been identified as HIV dependency factors (HDFs), which are necessary for efficient progression through the early and late stages of the viral life cycle [17,18,19,20,21]. HIV restriction factors (HRFs) are the host’s first line of antiviral defense, which primarily target the early steps of the viral life cycle. Although, already present in host cells and ready to inhibit viral replication at the time of infection, HRFs also serve as inducers of the innate immune response, which in turn further enhances the expression of HRFs [61,62].

In contrast to the multistep HIV-1 life cycle, the process of gene delivery by non-replicating HIV-1 vectors is a single round-of-infection event, which starts at vector attachment to the target cells and is completed with the initiation of transgene expression. In contrast to productive HIV life cycles, which result in the release of infectious viral particles, successful transduction by lentiviral vectors leads to long-term transgene expression and survival of the vector-transduced cells.

For more than a decade, various approaches have been employed by different groups to identify host factors affecting HIV-1 infection in vitro [63,64,65]. Interestingly, results from these studies demonstrated only a limited overlap. By default, the above studies could not identify candidate genes which contribute to the host in vivo environment and to the adaptive immune response to HIV-1 infection, which affect long-term expression of viral genes (e.g., HIV latency). Furthermore, in vitro screening methodologies, which are premised on supraphysiological expression levels of transgenes or highly efficient knockdowns of host gene expression, are probably not compatible with normal human physiology. Consequently, small molecules that efficiently target the above in vitro identified HDFs and HRFs are likely to inadvertently induce cytotoxicity, which limits their therapeutic value.

Small animal models using a single mouse strain are the premise of most safety and efficacy preclinical studies. The applicability of the results obtained in the current mouse models to human translational research has limitations and are probably mouse strain-specific. However, recent mouse models employing the EcoHIV chimeric HIV virus accurately emulated complex phenotypes observed in HIV infected patients, including HIV latency and HIV induced neurocognitive impairment [66,67]. Per contra, a cardinal study aimed at modeling the development of cellular immune response to AAV vector-transduced hepatocytes in gene therapy-treated patients did not exhibit characteristics of cellular immune response to AAV vector transduced hepatocytes in immunologically sensitized mice [68]. These findings were not in line with the outcomes of an earlier gene therapy clinical trial, in which hemophilia B patients treated with factor IX expressing AAV2 vectors exhibited a decline in therapeutic factor IX levels and an increase in hepatic transaminases [13]. Recently reported discrepancies between the stable long-term correction of factor VIII deficiency in preclinical and human clinical studies of AAV vector-based gene therapy for hemophilia A underscore the significance of this phenomenon [69]. The discrepancies between the above animal model and the outcomes of the AAV vector-based human clinical trial were attributed to several mechanisms, including differences in the function of mouse and human CD8 cells, as well as differences in mouse and human AAV capsid processing and antigenic presentation [68]. We raise the possibility that mouse-strain-specific traits could also contribute to the discrepancies between human clinical trials and their respective mouse preclinical studies, which are premised on a single mouse strain. Importantly, in the current study, we employed 42 genetically defined mouse strains to characterize the effects of the host genetic background on multiple parameters of hepatic gene delivery by lentiviral vectors. Interestingly, a comprehensive study by Uchil et al. demonstrated differences in the effects of mouse and human TRIMs on the HIV transduction efficiency of human and mouse cells in vitro. These finding suggest that an additional mechanism contributes the discrepancies between preclinical and clinical studies [70].

Throughout the study, we observed significant differences in hepatic transgene expression among the 41 CC mouse strains (e.g., a greater than 100-fold difference in hepatic luciferase expression in CC057 and CC061). Importantly, a wide phenotypic range across the CC mouse strain was common to all analyzed traits, including hepatic and splenic VCN and vector-specific activity. These findings indicate that the mouse genetic background, and by extrapolation, the genetic background of human patients, determine the efficacy and safety of preclinical and therapeutic lentiviral vector-based gene delivery protocols, respectively. We assert that significant variability in the outcomes of the currently employed gene therapy protocols should be expected. Also, it appears that the unexpected outcomes of gene therapy clinical trials cannot be predicted by the currently employed murine-based preclinical studies [68]. We assert that, in order to compensate for the limited ability to predict the outcomes of a gene therapy protocol in an individual patient, it is reasonable to consider the development of modified escalating dose-based protocols for non-fatal diseases.

In most CC mouse strains, periodic analysis of hepatic luciferase expression demonstrated stable or an increase in transgene expression between weeks 1 and 24 PVA. However, two CC mouse strains (CC021 and CC024) exhibited a continuous decrease in hepatic luciferase expression. The mean hepatic VCN of the CC021 strain (0.41 vector genomes per cell) was higher than the VCN of 24 CC mouse strains. Based on these findings, we theorized that the epigenetic silencing of lentiviral vectors carrying the hepatic specific promoter (hAAT) contributed to this phenomenon. This notion was supported by the fact that vector-specific activity (SA) in the CC021 hepatocyte was the lowest among the studied CC mouse strains. In contrast, the CC024 mouse strain hepatocytes exhibited the lowest mean VCN of 0.045 and an average SA, which suggested that either luciferase cytotoxicity or the host cellular immune response to the luciferase expressing hepatocytes were involved with the decline in hepatic luciferase expression between weeks 1 and 24 PVA [42]. The continuous decline in hepatic luciferase expression in CC021 and CC024 was mediated by two separate mechanisms. It took place in the context of healthy animals, which were transduced with integrating, self-inactivating (SIN) vectors carrying a liver-specific promoter. These characteristics suggest that the above mechanisms are probably part of a general host antiviral response, which is independent of the HIV LTR sequence, the vector integration site, and the nature of the vector’s internal promoter. These findings open new directions for identifying novel host factors that affect long-term expression from lentiviral vectors and could be involved in the establishment of latent HIV reservoirs. Furthermore, since the above phenomena were characterized in healthy mice, it is reasonable to assume that mild non-toxic alterations in the expression/function of the above newly identified host factors could be sufficient to prevent the decline in hepatic transgene expression from lentiviral vectors and to treat the latent HIV reservoirs, with minimal adverse effects. Interestingly, to a certain degree, the continuous decrease in luciferase in CC024 and CC021 is in line with the decrease in factor FVIII activity in hemophilia A patients treated with high doses of AAV vectors carrying factor VIII [69]. These findings suggest that the suitability of specific mouse strains to be used in preclinical studies is dictated by their genetic background. Furthermore, the choice of a specific mouse strain to serve in preclinical studies should be tailored to the investigated phenotype.

The notion that preclinical mouse studies can be employed to characterize complex, multifactorial pathologic traits in humans was the impetus to the development of the CC recombinant inbred mouse strains panel [71]. As described above, eight mouse strain were subjected to a carefully planned intercrossing breeding protocol, which generated novel, genotypically characterized mouse strains. The novel CC panel of mouse strains has been successfully employed in an increasing number of studies aimed at understanding the role of the genetic background in the development and outcomes of various pathologic processes. These include bacterial, fungal, and viral infections [24,25,72,73,74,75,76,77], metabolic disorders [78], inflammation [26], cancer [79,80,81], and neurological and immunologic disorders [82,83]. Here, we employed the valuable CC mouse strains panel to identify host factors that affect a therapeutic procedure.

The CC panel was designed to genetically characterize complex traits, including the identification of allelic combinations that quantitatively affect these traits. It was estimated that 1000 CC mouse strains would be required to meet the above objectives [71]. At this time, the merely 70 CC mouse strains currently available cannot provide the statistical power to accurately characterize highly complex traits. Although some of the above studies successfully associated QTLs (and at times, specific host factors) with specific phenotypes, the statistical power of the CC mouse panel was not sufficient to identify specific combinations of normal alleles that quantitively affected complex traits. Notwithstanding the statistical limitations of the current CC panel, it is a powerful research tool that provides novel insights that expand our understanding of biological pathways. The wide genetic variability within the CC panel accurately outlines the spectrum of strain-specific phenotypes and identifies specific CC mouse strains that exhibit the broadest strain-specific phenotypic divergence. This critical attribute facilitates further genetic characterization of the above phenotypes using F2-crossing studies (employing two phenotypically extreme strains). Furthermore, the current panel of CC mouse strains facilitates the identification of mouse strains which are most suitable to model therapeutic regimens in preclinical studies.

The current study aimed at identifying host factors that determine the ultimate outcome of hepatic gene delivery by the lentiviral vector, which was defined here as the mean of the hepatic luciferase expression level at week 24 PVA. The analysis of mouse strain genotypes and hepatic luciferase expression failed to identify a QTL that associates with hepatic luciferase expression at week 24 PVA. As outlined previously, successful hepatic transduction, which results in long-term transgene expression, is a multistep process. Each step in this process is affected by multiple HRFs and HDFs, which are encoded by eight different alleles. Unless a highly potent allele is included in the genetic pool of the eight CC founders, it is likely that the statistical power of a 41 CC mouse strain-based genetic study will not be sufficient to associate a QTL with hepatic luciferase expression at week 24 PVA.

To circumvent the above statistical power limitations in the current panel of CC mouse strains, we sought to narrow the complexity of the studied phenotypes. To this end, we defined the relative differences in hepatic luciferase expression between weeks 1 and 3 PVA (normalized by the level of luciferase expression at week 1 PVA (Luc-W3–Luc-W1/Luc-W1)) as a new trait, which exhibited the highest correlation with hepatic luciferase expression at week 24 PVA. This approach is premised on the notion that in contrast to the multitude of host factors that affect multiple steps in the process of hepatic lentiviral transduction, a smaller number of host factors are involved in the mechanism that affects the kinetics of hepatic transgene expression between weeks 1 and 3 PVA. We theorized that the narrowed trait complexity would increase the likelihood of associating a QTL with the phenotype of interest. Indeed, a QTL in chromosome 1 was associated with an increase in hepatic luciferase between weeks 1 and 3 PVA. The 129s1/SvImJ founder allele in the above QTL was shared by five CC mouse strains which exhibited the highest increase in hepatic luciferase expression between weeks 1 and 3 PVA. Within the above QTL, we identified four candidate genes, which can potentially contribute (individually or synergistically) to the studied trait. These genes include *SERTAD4*, *IRF6*, *TRAF3IP3*, and *UTP25*, which are involved in embryo development, cancer, innate immune response, and epigenetic processes [49,50,52,54]. Interestingly, the impact of *TRAF3IP3* on interferon pathways [54] could be in line with earlier reports demonstrating the effects of interferon on hepatic lentiviral vector transduction [42]. The relative proximity of the above genes in chromosome 1 raises the possibility that their expression is coregulated. Interestingly, findings from a recent study indicated that genetic variants in either the *UTP25* or the *IRF6* promoters affect their interactions with either *IRF6* and *TRAF3IP3* or *UTP25* and *TRAF3IP3*, respectively [60]. These interactions exerted different effects on the expression level of the above genes. Further research is required to determine the effects of each of the above candidate genes on lentiviral transduction.

During this study, we observed phenotypic variability among mice of the same strain (isogenic-phenotypic discordance). The level of the observed isogenic discordance, (quantified as coefficient variation) was mouse strain- and phenotype-dependent. Phenotypic discordance between monozygotic twins has been associated with several human diseases [84]. The phenomenon of isogenic discordance has been mechanistically studied in different animal models [85,86]. One of the first murine models studying isogenic discordance was premised on mice carrying the *agouti viable yellow (A^vy^)* allele [87,88]. *A^vy^* mice exhibit various fur colors, ranging from agouti to complete yellow, which are secondary to the expression level of the agouty protein (yellow and agouti fur colors are associated with high and low levels of the agouti protein, respectively). This phenomenon is attributed to germline insertion of an intracisternal A-type particle (IAP) transposon upstream to the agouti protein coding sequence. Consequently, transcriptional regulation of the *A^vy^* allele is mediated by the IAP LTR, whose methylation inversely correlates with its transcriptional activity and varies among isogenic *A^vy^* mice. Isogenic alleles, which demonstrate variable epigenetic profiles and consequently, different transcriptional activity, are termed metastable epialleles [89,90,91].

The unique DNA methylation patterns of IAP LTRs are established during gametogenesis and early embryogenesis, which involve epigenetic reprograming processes, including two waves of genome-wide DNA de-methylation (in primordial germ cells and preimplantation embryos), followed by de novo methylation [92,93,94,95]. Partial methylation of IAP LTRs in the preimplantation embryo and during gametogenesis indicate that the IAP LTRs are relatively resistant to the above genome-wide demethylation processes [94,95,96]. Early publications suggested that DNA hypermethylation, which represses the expression of IAPs and other retrotransposons (especially during gametogenesis), is part of a host defense mechanism that minimizes genomic instability due to mutagenic germ line insertions of retro-elements [97,98]. On a cellular level (which is mostly mouse strain-independent), the phenomena of genomic imprinting and chromosome X inactivation in female cells result in allele-specific gene expression. Interestingly, an epigenetic modifier that affects the expression of imprinted genes also associates with IAP genomes [47]. Furthermore, the fur color of *A^vy^* mice is affected by the maternal phenotype, which is a genomic imprinting characteristic [44,88,90].

Most IAP genomes are transcriptionally silenced by suppressive histone modifications and DNA methylation [89,94,96,99]. However, specific IAP proviruses show variable levels of DNA methylation and maintain variable transcriptional activity in a locus-specific manner in isogenic mice. In IAP-comprising metastable alleles, the process that determines the methylation status of a specific IAP is stochastic. However, the overall methylation status of a metastable allele in specific mouse strains is probabilistic and affected by several variables, including the IAP sequence, the IAP integration site, and the mouse strain genetic background [100,101,102], which determines the strain-specific expression profile of multiple epigenetic modifiers [103,104,105]. Importantly, KRAB-ZFPs, which play a major mechanistic role in the epigenetic repression of IAP transcription, have been evolved to target strain-specific IAP sequences [89,106]. Several studies reported environmental effects on the epigenetics and gene expression of metastable epialleles. These include nutritional alteration, exposure to ionizing radiation, heavy metals, ethanol, and bisphenol A (BPA) [107,108,109,110,111]. However, later studies could not fully support some of the above earlier reports [112,113].

Notwithstanding the central role of IAP sequences in the metastable epiallelic phenomena, the Whitelaw team described the variegation of transgene expression from tandems of expression cassettes lacking IAP sequences [114,115]. The above transgenic construct contained the green fluorescence protein (GFP) open reading frame under the control of the human a-globin promoter. Variegation levels of erythrocyte GFP expression were mouse clone-specific. However, the intra clone variability of the variegation level (between mice within a specific mouse clone) has not been reported. Furthermore, using a germ line chemical mutation procedure, the Whitelaw team identified secondary epigenetic modifiers which either enhanced or suppressed clonal variegation and altered the expression of bona fide metastable epialleles in *A^vy^* mice [114]. These findings suggest that unique *cis* elements, such as IAP sequences and imprinting control elements (ICE), are the primary initiators of the metastable epigenetic phenomenon, which is premised on local epigenetic permutations at the time of genome-wide epigenetic reprograming processes during either gametogenesis, preimplantation of the embryo, and probably, during some terminal differentiation processes [116,117,118,119]. It is likely that the association of specific trans elements such as KRAB-ZFPs with the above primary *cis* elements is an early (and probably, a required) event in the establishment of metastable epiallelic hubs [47,120,121]. This early event is followed by interactions with the above secondary epigenetic modifiers, whose levels affect the range of metastable allele expression (and DNA methylation) among isogenic mice [114]. Per contra, genome-wide epigenetic reprograming processes during cellular differentiation [116,117,118,119] can potentially result in the variegation of transgene gene expression, whose range is also determined by the above epigenetic modifiers described by Blewitt et al. [114]. It is possible that during genome-wide epigenetic changes (typical of gametogenesis, early embryo development, and cellular differentiation), a balance between two pathways, including cellular differentiation (premised on DNA demethylation; CTCF dependent) and repression of the transposable element (premised on epigenetic transcriptional repression, requiring KRAB-ZFPs) results in isogenic phenotypic discordance. In this study, we observed phenotypic-isogenic discordance in several parameters of hepatic transduction by lentiviral vectors. In contrast to earlier transgenic mouse models, the lentiviral vector genomes in this study did not pass through gametogenesis or early preimplantation embryonic processes. Furthermore, the integration site profile of the lentiviral vectors is semi-random [122]; thus, the likelihood that a significant number of lentiviral vectors integrated in proximity to the IAP sequences is low. Note that the self-inactivating (SIN) lentiviral vectors in this study were devoid of the HIV U3 region (which comprises the parental HIV enhancer–promoter sequences). Interestingly, endogenous lentiviral sequences were identified in rodent and primate genomes [123,124,125,126]. Furthermore, murine proteins show homology to HIV Gag [127,128]. Based on these facts, we cannot dismiss the theory that lentiviral vector sequence-directed KRAB-ZFPs contributed to the intrastrain phenotypic variations observed in this study. Also, we cannot entirely rule out the possibility that KRAB-ZFPs directed to murine retroelements cross react with the lentiviral vector genomes. Since mouse hepatic transduction by lentiviral vectors is highly regulated by HRFs and HDFs, which also regulate the life cycle of murine endogenous and exogenous retroviruses, we raise the possibility that variation in the expression levels of the above host factors in isogenic mice probably contributed to the intrastrain phenotypic variations which were characterized in this study. The large number of the host factors involved in the various steps of lentiviral vector hepatic transduction and the number of mouse-strain-specific transposable elements (TE) in the mouse genome support the above notion [45]. The wide range of mouse-strain-specific CVs of multiple lentiviral vector transduction traits suggests that the phenomenon of isogenic phenotypic discordance is not rare. To characterize the role of host genetics in the mechanism of phenotypic variability in isogenic mice, an LOD score analysis was employed to identify two QTLs. A QTL of 9.28 Mb (comprising 372 and 134 genes and pseudogenes, respectively) in chromosome 7 was associated with the CV of hepatic luciferase expression at week 3 PVA. A second and significantly narrower QTL of 1.61 Mb in chromosome 4 was associated with the CV level in the ratio of liver and spleen VCNs. Specifically, increased CV levels were associated with the 129s1/SvImJ allele in the above QTL in three CC mouse strains. Premised on an earlier study [45], further QTL characterization identified eight 129s1/SvImJ-specific insertions of retroelements, including five SINEs, two RLTR10s, and a single MaLr insertion, which was located within a cluster of eight CTCF target sequences. The central role of CTCF and its interactions with transposable elements in the phenomena of phenotypic variation in isogenic mice and in genomic imprinting, as well as in strain-specific variation in gene expression levels, has been reported earlier. Although the 129s1/SvImJ allele in the above QTL was associated with the highest CV, it does not contain strain-specific [43,44,45,46] IAP insertions, whose variable methylation (VM) status has been mechanistically associated with the phenomenon of metastable epialleles [43,87,88]. Importantly, although not common, VM of non-IAP retroelements has been reported by recent studies [43,44]. However, their effect on intrastrain variation in gene expression has not been documented. The studied phenotype (CV of splenic VCN/hepatic VCN) was premised on lentiviral VCN in two tissues. There is a possibility that the expression of a host factor affecting VCN exhibits intrastrain variability in a tissue-specific manner. This phenomenon was described in an earlier report regarding the tissue-specific variable methylation of IAP sequences (tsVM-IAP) in isogenic animals [43]. However, the tsVM of non-IAP retroelements and its effect on gene expression has not been reported. Note that the human genome is devoid of IAP sequences. Thus, the phenomenon of metastable epialleles in humans [91,129] is likely to involve non-IAP transposons.

The ultimate dependency of viruses on host physiologic and metabolic pathways, which are probably modulated by the host circadian rhythm [130,131], conjure up the notion that host sleep patterns affect lentiviral vector transduction efficiency. An earlier study demonstrating major circadian rhythm effects on influenza A virus (IAV) and herpes simplex virus 1(HSV-1) infection in vivo [132] supported the above notion and was the main impetus to correlate sleep patterns of CC mouse strains with overall lentiviral vector transduction efficiency, as determined by hepatic luciferase expression levels at week 24 PVA. This study describes, for the first time, a moderate correlation between behavioral/sleep patterns and in vivo lentiviral vector transduction efficiency. Our findings suggest that the host sleep pattern is one of various host physiologic pathways that determine the outcomes of lentiviral vector transduction. Furthermore, as outlined by Sulli et al. [130], the circadian rhythm has relatively modest effects on gene expression. Two mechanisms can separately, or in combination, contribute to this phenomenon. One mechanism requires that host factors affecting sleep patterns are also involved in the multistep process of hepatic transduction. However, there is also a possibility that the maternal behavior/sleep pattern from the time of gametogenesis to the progenies preweaning period can epigenetically induce lifelong alterations in gene expression, which affect permissiveness to lentiviral vector transduction [86,133,134,135]. Note that notwithstanding the circadian rhythm effects on multiple physiologic pathways and the outcomes of viral infections [130,131,132,136,137], mouse strains with altered melatonin function are the premise of animal models in basic research and preclinical studies [138,139]. Furthermore, animal care in most academic animal facilities is conducted during the mouse daylight/sleep period. The animal care-induced sleep disturbances can be considered as an environmental factor, whose effects on mouse sleep patterns are mouse strain-dependent, which can contribute to the phenomenon of intrastrain phenotypic differences.

## 5. Clinical Relevance

The overall goal of this study is to facilitate the broadening of the disease spectrum for the gene therapy approach to non-fatal diseases. Current lentiviral gene therapy protocols involving HSPC and circulating T cells target fatal diseases. Based on established risks/benefits considerations, the FDA approved the above therapeutic protocols. Recent reviews, which demonstrated the success of HSPC and circulating T cell-based gene therapy protocols, alleviated some biosafety concerns and strongly supported the above FDA policies [140,141,142,143,144,145]. Clearly, the considerations and conclusions outlined in this study are directed to gene therapy protocols for non-fatal diseases. We assert that patient-to-patient variations and discrepancies between mouse models and clinical trials bound the lentiviral vector-based gene therapy system to fatal diseases. The characterization of gene therapy applications in multiple mouse strains may alleviate the above limitations and biosafety concerns.

Furthermore, novel host factors affecting viral vector gene delivery could potentially serve as biomarkers for future personalized gene therapy protocols. Interestingly, recent studies indicated that limitations in animal models and mouse strain-specific variations are not limited to lentiviral vector-based gene therapy and are probably shared by conventional therapeutics [146,147].

Note that clinical trials using HSPC demonstrated discordance with preclinical studies. Aiuti et al. [148] described the development of an optimal viral vector and therapeutic protocols which were premised on clinical trials. These improvements could have been established following preclinical studies. Cavazzana-Calvo et al. [9] reported a loss of polyclonality in a successful gene therapy protocol for b-thalassemia. This phenomenon was not reported by earlier preclinical trials. Based on the work of Zhao et al. [7], one could theorize that the above adverse effect could have been identified in multi-strain-based preclinical trials. The discrepancy between AAV vector-based preclinical and clinical trials of hemophilia A were described by Schutgen et al. [69]

This study confirmed the hypothesis that mouse-strain-specific host genetic backgrounds determine the mouse-strain-specific phenotypes/parameters of hepatic lentiviral vector transduction. Since transduction efficiency by all viral vectors is totally contingent upon host-dependent and restriction factors, we assert that the above conclusion is applicable for all viral vectors, regardless of vector design and mode of vector administration. Clearly, all the phenotypes studied in a preclinical trial should be specifically tailored to the relevant clinical trial. A specific viral vector will demonstrate specific transduction efficiency in a cell line and a mouse-strain-specific fashion. Thus, the importance of employing multi-strain preclinical studies is relevant to all preclinical applications, including those evaluating conventional medications [147].

The effects of the host genetic background on the safety and efficacy of vector-delivered therapeutic transgenes have not been investigated in this study, which focused on the gene delivery aspect of the lentiviral vector system.

We assert that transgene safety and efficacy phenotypes, including vector immunogenicity, vector-specific activity, and toxicity, are determined by the host genetic background. Thus, we expect mouse-strain-specific differences in the above parameters.

Codon optimization of vector-delivered cDNA is a common practice employed to increase vector-specific activity. However, co-translational modifications and protein folding of codon-optimized cDNAs are host factor-dependent and thus, can increase the phenomenon of mouse strain-to-mouse strain phenotypic differences [149]. The gene editing approach to correct genetic mutations potentially reduces transgene expression-related mouse strain-to-mouse strain variations and by inference, patient to patient phenotypic differences. On the other hand, the dependence of gene editing on host DNA repair pathways can further contribute to the above phenomenon.

Based on the efficacy and safety data from clinical trials, the FDA rightly approved CAR T cells as gene therapy anti-cancer medications [144]. Notwithstanding the ultimate therapeutic successes of CAR T cells, we raise the possibility that additional multi mouse-strain studies can further improve CAR T cells protocols. Specifically, genetically defined mouse strains can facilitate the identification of host factors involved in cytokine storms and susceptibility to tumor development. These genes can serve as predictive markers for therapeutic efficacy and the development of adverse effects. In addition, the above multiple mouse strain-based studies may suggest that the doses of administered CAR Ts can also be adjusted to the total number of administered integrated lentiviral vector genomes.

## 6. Conclusions

In this study, 41 genetically characterized CC mouse strains were employed to investigate the effects of the host genetic background on hepatic transduction by lentiviral vectors.

The study demonstrated a wide range of mouse strain-specific differences in various aspects/traits of lentiviral vector hepatic gene delivery. These major host genetic background effects on lentiviral vector transduction in vivo suggest that novel multiple mouse strain-based approaches of evaluating the efficacy and safety of viral vector-based gene delivery in preclinical studies of non-fatal diseases should be considered. We believe that these new approaches will narrow the current discrepancies between the outcomes of preclinical and clinical studies and will be highly cost-effective.

The genetic diversity of human patients, which is not narrower than the genetic diversity of 41 CC mouse strains, explains the expected patient-specific outcomes of viral vector-based gene therapy protocols for non-fatal diseases. We assert that accumulating or escalating dose-based protocols of viral vector administration should be considered for gene therapy protocols for non-fatal diseases.

We used the CV of various gene delivery phenotypes associated two QTLs with intrastrain phenotypic variations. These findings suggest that the phenomenon of phenotypic isogenic discordance in mice is not extremely rare and can be further characterized using large numbers of genetically defined mouse strains.

In line with earlier reports [132], this study correlated, for the first time, the efficiency of lentiviral transduction in vivo with strain-specific sleep patterns. The findings of this study raise the possibility that routine mouse care procedures in most animal facilities induce sleep disturbances, with strain-specific effects on the outcomes of preclinical studies.

## Figures and Tables

**Table 1:** Summary statistics for the mean of each phenotype across C57B6 and 42 Collaborative Cross (CC) mouse strains. The table outlines the minima (lowest value), first quantile (25th percentile), median, mean, third quantile (75th percentile), and maxima (highest value) of the calculated means across all mouse strains. SA is vector specific activity. It is measured as the ratio of luciferase expression at week 24-PVA and expressed as photon/s/vector-genome/cell.

	Luc W1 photon/s	Luc W3 photon/s	Luc W6 photon/s	Luc W8 photon/s	Luc W14 photon/s	Luc W24 photon/s	$\frac{W3-W1}{W1}$	$\frac{W8-W3}{W3}$
Min	960,125	670,875	614,943	640,300	978,000	1,016,900	−0.625	−0.158
First Quantile	4,735,500	4,040,833	5,517,083	4,660,000	5,528,750	5,524,792	0.036	0.140
Median	7,154,583	12,271,667	14,277,083	12,921,700	14,581,267	14,131,083	0.421	0.308
Mean	16,051,377	22,468,382	25,291,655	26,118,529	26,426,337	25,063,259	0.519	0.386
Third Quantile	19,337,500	22,962,083	30,437,188	30,902,917	35,147,708	32,247,500	0.984	0.617
Max	96,837,500	177,962,500	163,275,000	178,150,000	193,912,500	130,412,500	2.150	1.619
	$\frac{W14-W8}{W8}$	$\frac{W24-W14}{W14}$	$\frac{W24-W3}{W3}$	$\frac{W24-W1}{W1}$	**Liver VCN** **Vector-genome/Cell**	**Spleen VCN** **Vector-genome/Cell**	$\frac{Liver\;VCN}{Spleen\;VCN}$	**Vector SA** **(Specific Activity)**
Min	−0.419	−0.522	−0.481	−0.158	0.045	0.039	0.434	6,897,779
First Quantile	−0.058	−0.099	0.148	0.140	0.256	0.108	1.809	21,511,854
Median	0.020	0.093	0.467	0.308	0.387	0.159	3.053	41,832,255
Mean	0.088	0.156	0.461	0.386	0.471	0.194	3.665	60,354,896
Third Quantile	0.234	0.267	0.726	0.617	0.602	0.218	4.329	84,925,752
Max	0.627	1.220	1.561	1.619	1.911	0.783	12.175	228,820,708

**Table 2:** Summary statistics for the coefficient of variation (CV) of each phenotype across C57B6 and 42 Collaborative Cross (CC) mouse strains. The table outlines the minima (lowest value), first quantile (25th percentile), median, mean, third quantile (75th percentile), and maxima (highest value) of the calculated CV across all mouse strains. CV = Standard Deviation/Mean × 100%. SA is vector specific activity. It is measured as the ratio of luciferase expression at week 24 PVA and expressed as photon/s/vector-genome/cell.

	Luc W1	Luc W3	Luc W24	Liver VCN	Spleen VCN	$\frac{Liver\;VCN}{Spleen\;VCN}$	Vector SA (Luc W24/VCN)
Min	20.77	35.63	18.51	19.33	14.81	4.38	20.89
First Quantile	46.87	52.04	50.02	32.54	27.44	40.26	50.43
Median	57.68	61.48	64.75	45.54	39.63	51.26	65.33
Mean	70.81	72.54	72.13	49.01	43.66	54.26	71.02
Third Quantile	98.57	96.20	90.83	64.62	51.40	63.37	79.82
Max	155.21	184.08	159.87	95.44	160.22	135.20	165.26

**Table 3:** Phenotypes global heritability. For each phenotype that has replicated samples within each CC line, we perform one-way ANOVA analysis where the CC lines are modeled as a categorical variable. The corresponding *p*-value in testing the CC effect and the global heritability which is calculated as the percentage of the phenotype variance explained by the CC lines are summarized.

Phenotype	Heritability (%)	*p*-Value
Liver Luc Week 1	44.94	<0.0001
Liver Luc Week 3	49.20	<0.0001
Liver Luc Week 6	43.19	<0.0001
Liver Luc Week 8	49.51	<0.0001
Liver Luc Week 14	50.28	<0.0001
Liver Luc Week 24	45.45	<0.0001
$\frac{Liver\;Luc\;Week\;3-Week\;1}{Week\;1}$	35.60	<0.0001
$\frac{Liver\;Luc\;Week\;8-Week\;3}{Week\;3}$	23.01	0.0370
$\frac{Liver\;Luc\;Week\;14-Week\;8}{Week\;8}$	14.79	0.6819
$\frac{Liver\;Luc\;Week\;24-Week\;14}{Week\;14}$	15.79	0.6130
$\frac{Liver\;Luc\;Week\;24-Week\;3}{Week\;3}$	18.92	0.3018
$\frac{Liver\;Luc\;Week\;24-Week\;1}{Week\;1}$	43.44	<0.0001
Liver VCN	59.02	<0.0001
Spleen VCN	53.77	<0.0001
Liver VCN/Spleen VCN	54.28	<0.0001
Liver Luc Week 24/Liver VCN	49.03	<0.0001

**Table 4:** QTL analysis of hepatic lentiviral vector traits. For each phenotype, QTL analysis is performed, and the genome wide significance is estimated based on the result from total 1000 permutations. Phenotypes with top candidate loci that passed the genome wide significance at 0.2 are summarized. The chromosome and position (Mb) refer the estimated QTL location followed by the lower and upper bounds of the 95% Bayes credible interval and the wide of the QTL interval. The reported heritability of each QTL corresponds to the estimated heritability of the top SNP associated with the reported QTL. The numbers of genes (coding and non-protein coding) and pseudogenes located within the 95% Bayes credible interval are also reported for the three QTLs that pass the 0.05 genome wide significance level. * For each outcome, if QTL analysis is performed not on the individual measurements, but on the mean or CV of each CC line, the corresponding phenotype is referred as phenotype mean or phenotype CV, respectively.

Phenotype *	Sig	*p*-Value	LOD	Chr	Position (Mb)	Lower Bound (Mb)	Upper Bound (Mb)	Interval Width (Mb)	Heritability (%)	# of Genes	# of Pseudogenes
CV. $\frac{Liver\;VCN}{Spleen\;VCN}$	0.05	0.008	9.47	4	135.41	134.82	136.43	1.61	65.48	57	11
CV. Liver Luc Week 3	0.05	0.030	8.92	7	50.19	40.97	50.25	9.28	63.26	372	134
$\frac{Liver\;Luc\;Week\;3-Week\;1}{Week\;1}$	0.05	0.040	8.37	1	192.69	192.36	193.71	1.35	60.92	34	4
CV. Vector SA (Liver Luc Week 24/Liver VCN)	0.10	0.064	9.72	8	99.03	99.03	100.59	1.56	66.45		
Liver Luc Week 1	0.10	0.081	11.27	2	154.29	152.95	156.38	3.43	71.79		
Liver VCN/Spleen VCN	0.10	0.091	9.86	4	19.80	14.75	19.80	5.05	66.95		
Liver Luc Week 6	0.20	0.143	10.12	1	37.56	37.54	41.37	3.83	67.91		
$\frac{Liver\;Luc\;Week\;14-Week\;8}{Week\;8}$	0.20	0.159	7.34	8	36.09	31.11	61.36	30.25	56.16		
CV. Spleen VCN	0.20	0.166	11.90	19	27.49	24.77	28.06	3.29	73.72		

## Data Availability

All the data used in this study are provided in the article and in the Supplementary Materials section.

## Supplementary Materials

The following supporting information can be downloaded at: https://www.mdpi.com/article/10.3390/v17020276/s1. Table S1: Raw data of all phenotypes.
